# Do Estimates of Women’s Control over Income and Decisionmaking Vary Across Nationally Representative Survey Programs?

**DOI:** 10.1007/s11205-025-03605-x

**Published:** 2025-04-25

**Authors:** Kalyani Raghunathan, Mai Mahmoud, Jessica Heckert, Gayathri Ramani, Greg Seymour

**Affiliations:** 1 Poverty, Gender and Inclusion Unit, International Food Policy Research Institute, New Delhi, India; 2 School of Business, Abdul Latif Jameel Poverty Action Lab (J-PAL)-MENA at the American University in Cairo, Cairo, Egypt; 3 Poverty, Gender and Inclusion Unit, International Food Policy Research Institute, Washington, DC USA; 4 (formerly) Nutrition, Diets, and Health Unit, International Food Policy Research Institute, Washington, DC USA; 5 US Census Bureau, Washington, DC USA

**Keywords:** Women’s empowerment, Decisionmaking, Control over income, Survey design, Africa, Asia

## Abstract

Empowering women is an explicit aim of Sustainable Development Goal (SDG) 5 and underpins 12 of the 17 SDGs. It is also a key objective of other pan-national agreements, such as the Comprehensive African Agriculture Development Programme. Tracking global progress toward these goals requires being able to measure empowerment in ways that are consistent and comparable—both within and across countries. However, empowerment is a complex concept, hard to quantify, and even harder to standardize across contexts. Two large survey programs—Feed the Future and the Demographic Health Surveys—ask women about two aspects of empowerment, their control over income and input into decisionmaking. Each program uses a different set of questions administered to different sub-populations of women. We use data from 12 countries to show that large within-country inter-survey differences persist even after efforts to harmonize questions and samples. Where available, we compare the FTF and DHS with the Living Standards and Measurement Surveys-Integrated Surveys on Agriculture. We present several hypotheses related to survey structure and survey administration to explain these inter-survey differences. We then either test for or rule out the role of these competing theories in driving differences in levels and in associations with commonly used characteristics. Standardizing survey measures of decisionmaking and control over income and how they are administered is important to track progress toward the SDGs; meanwhile, caution should be exercised in comparing seemingly similar survey items across survey programs.

**Supplementary Information:**

The online version contains supplementary material available at 10.1007/s11205-025-03605-x.

## Introduction

Adopted by United Nations Member States in 2015, the Sustainable Development Goals (SDGs) set ambitious 2030 targets of eradicating poverty and hunger, ensuring access to healthy lives, quality education, sanitation, and livelihoods, and promoting planetary health through more responsible production and consumption practices. Gender equality and the empowerment of women and girls (SDG 5) is a standalone aim that also underpins indicators for 12 of the 17 SDG goals (Pryor & Seck, 2019).

Women’s empowerment, defined by Kabeer as the “ability to make strategic life choices where this ability was previously denied” (Kabeer (1999): 437), is a fundamental human right, and also facilitates improvements in other aspects of wellbeing like maternal and child health and nutrition, household food security and child education in multiple country contexts (Amugsi et al., 2016; Carlson et al., 2015; Cunningham et al., 2015; Heckert et al., 2019; Sraboni et al., 2014; Van den Bold et al., 2013). Monitoring progress towards enhancing women’s empowerment and gender equality requires investments in measuring these outcomes and collecting sex-disaggregated data; however, current gaps in both the availability of data and in data quality undermine these efforts (Heckert & Fabic, 2013; Pryor & Seck, 2019; Richardson, 2018).

Measuring empowerment is not straightforward; it is a complex, multidimensional, and context-dependent concept that has been measured in many different ways (Agarwala & Lynch, 2006; Kabeer, 1999; Miedema et al., 2018; Santoso et al., 2019). In this paper, we focus on one aspect of empowerment—related to what Salcedo et al. (2024) call “material environment”—comprising women’s control over the use of income from various sources and their participation in decisions on large household purchases. These are important and commonly used indicators of women’s instrumental agency; an inherently difficult aspect to measure (Desai et al., 2022), but one that forms the “essence of empowerment” (Malhotra et al., 2002). Many multitopic surveys that have limited space to allocate to women’s empowerment, distill the concept down to focus on these two indicators, and related questions have been included in many surveys. We use data from nationally representative survey programs across 12 countries in South and South-east Asia, Central America and the Caribbean, and East and West Africa to study how variation in survey questions, design, and administration can affect both the levels of women’s material environment and its association with individual and household characteristics.

Despite the context specific nature of women’s empowerment, efforts to develop indicators to track gender equality and women’s empowerment and enable cross-country comparisons have been ongoing for almost three decades (Buvinic et al., 2020; Charmes & Wieringa, 2003; Malapit et al., 2019; Salcedo et al., 2024; Schmid, 2022; Seymour et al., 2024). Many of these indices are conceptually grounded in feminist contributions, and some—notably the African Gender Equality Index or AGDI (Charmes et al., 2023)—have taken great care to develop indicators that are culturally relevent. Although valuable viewpoints argue the advantages of context specific indicators over universal ones (Richardson, 2018), a clear need remains for empowerment indicators that can be used across contexts. Decisions over large purchases and control over income define empowerment narrowly, however, they are commonly used and embedded in widely adopted targets. For example, control over income and decisions on large purchases relate closely to two of the SDG-5 targets, Target 5.5 and Target 5.a. In addition, member states under the Africa-wide Comprehensive African Agriculture Development Programme (CAADP) have committed to increasing the “proportion of rural women that are empowered in agriculture” by 20% by 2025; two of the five domains, to be assessed biennially, are decisionmaking and control over use of income (African Union, 2021). Efforts to monitor CAADP indicators primarily rely on existing data collection efforts. Even large survey programs cover a limited number of countries and are not collected frequently enough for biennial reporting, thus prompting the question of whether combining results across multiple survey programs is a reasonable solution.

The main survey programs we consider are the well-known and nationally representative Demographic Health Surveys (DHS), and the Feed the Future (FTF) surveys, representative of large portions of countries. The two survey programs have different objectives: the DHS focus on population, health, and nutrition themes while the FTF surveys focus on agriculture and food security. Both view women’s empowerment as an integral part of their overall objectives and ground their approach to measuring women’s empowerment in the “ability to make strategic life choices” (Kabeer (1999): 437). The DHS first included women’s empowerment as part of an optional women’s status module in 1995 and it has been part of the core women’s questionnaire since 2009 (Heckert & Fabic, 2013; Kishor & Subaiya, 2008). The FTF surveys integrated women’s empowerment since their inception in 2012, and the Women’s Empowerment in Agriculture Index (WEAI) was designed specifically for FTF as a comprehensive measure of women’s empowerment (Alkire et al., 2013). Where possible, we also show comparisons with the Living Standards and Measurements Study-Integrated Surveys on Agriculture (LSMS-ISA), led by the World Bank in collaboration with national statistical agencies and other partners, though this survey is only available for three countries in our sample. We consider similar populations at similar points in time and harmonize our outcome measures. In selecting comparable rounds of data and units within each dataset, we pay careful attention to geography and survey design, including stratification, primary sampling units, and respondent selection. Despite these efforts, we document substantial differences across surveys in our two measures of women’s empowerment, and in their associations with individual and household characteristics. We examine potential sources of these differences, with specific attention to biases introduced by aspects of survey design and administration.

We then draw on the existing literature to present hypotheses for the large and significant differences in the levels of control over income and decisionmaking indicators between surveys within countries. Where possible, we either test these hypotheses descriptively or include controls for them in our analyses of the association of our two measures of material empowerment with key individual and household characteristics. Cross-country comparisons are not the focus of our paper; instead, we provide insights into the process of inference about aspects of women’s empowerment based on different data sources for very similar samples within the same geography.

Several papers in the literature are closely related to ours. Most recently, Salcedo et al. (2024) use the DHS surveys for 45 low- and middle-income countries and the Alkire-Foster method (Alkire & Foster, 2011) to propose the Multi-dimensional Women’s Empowerment Index, an additive, decomposable index with eight indicators, two each in the domains of health, material environment, social relationships and physical integrity. The two indicators in the material environment domain are the same as the ones we use here, insufficiencies in which account for about a quarter of the disempowerment in the 15 least empowered countries (Salcedo et al., 2024; Fig. 3). Miedema et al. (2018) use DHS data from five countries in East Africa to investigate domains of empowerment. Their analysis confirms a three-domain model of empowerment, one of which is participation in household decisionmaking, and identifies a subset of items that can be used to monitor progress towards women’s empowerment related goals. Using DHS data from 34 African countries, Ewerling et al. (2017) propose the Survey-based Women’s EmPowERment index (SWPER) which permits comparisons both across countries and within countries over time. Here too, decisionmaking emerges as one of the three domains of empowerment from the authors’ principal component analysis.^1^ Finally, Peterman et al. (2021) use household survey data from Ecuador, Yemen and Uganda to investigate what decisionmaking questions actually capture, and how they are related to other common proxies for women’s empowerment in the literature, such as education. They show that question framing and variations in indicator construction can lead to substantial variations in measures of empowerment across households and call for more in-depth country-specific analyses into the process of decisionmaking and measurement using household surveys.

Our paper builds on and extends this literature by showing that descriptive results for control over income and decisionmaking can differ widely for very similar samples within a single country, and that even after correcting for survey administration and survey design elements as far as possible, associations of these measures of women’s empowerment with commonly used individual and household characteristics differ in both size and direction. In doing so we hope to refocus attention on the tricky issue of quantifying women’s empowerment and to encourage future efforts to improve existing measures.

## Data

The FTF and DHS programs were selected for three main reasons. First, these are standardized surveys covering multiple countries. Second, they are large-scale surveys designed to be representative, if not nationally, then of large regions within countries. Third, both collect data on control over use of income and household decisionmaking, permitting us to construct comparable outcome variables. The third survey program we consider, the LSMS-ISA, is only available for Malawi, Tanzania and Uganda, so we relegate the discussion of the LSMS-ISA’s sampling strategy and respondent selection to the Online Resource.

### Sample

In selecting survey rounds within a country, we chose those that were conducted as close as possible to one another, to limit differences attributable to change over time. Since the FTF surveys have only been conducted once or twice per country, the DHS rounds were selected based on their proximity to the most recent FTF survey year. With some exceptions, we used all FTF data that was publicly available when this work commenced in May 2020.^2^ Table 1 reports the survey year, sample frame and sample size for each country and survey used in this paper.

**Table 1:** FTF and DHS survey characteristics by country

Country	FTF			DHS		
	Month and Year	Sample frame	Post-harmon-ization sample size	Month and Year	Sample frame	Post-harmon-ization sample size
Bangladesh	January–June, 2015	Rural areas of each of the 7 administrative divisions of the country	4207	June–November, 2014	7 administrative divisions	7708
Nepal	August–September 2015	20 districts in 3 regions	592	March–October 2016	7 provinces	1663
Cambodia	August, 2015	4 provinces	899	June–November, 2014	14 provinces and 5 groups of provinces	1457
Ghana	July, 2013	3 northernmost regions, plus several areas in the Brong-Ahafo Region	2860	September–December, 2014	10 regions	2060
Malawi	July–October 2013	7 districts in 2 regions	694	October 2015- February 2016	28 districts	3024
Mozambique	January–April 2013, November 2013–January 2014	23 districts in 4 provinces	316	May–December 2011	11 provinces	2718
Kenya	May–June, 2015	9 counties in northern, western, and southern Kenya	1183	May–October, 2014	47 counties	2746
Tanzania	May–July, 2016	8 regions	507	September 2015- January 2016	30 regions (including Dar as Salam city)	1659
Uganda	March–May, 2015	38 districts	504	June–December, 2016	15 regions, the greater Kampala area, the island districts and the mountain districts	3998
Rwanda	December 2014- January 2015	27 districts	764	November 2014- April 2015	30 districts	5693
Haiti	October–December, 2012	3 Corridors (Northern, Marc, and Cule-de-Sac)	413	January–June, 2012	10 departments	2583
Honduras	June–August, 2012	6 departments in western Honduras	1893	October–2011- June 2012	18 departments	2615

FTF: Feed the Future, DHS: Demographic Health Surveys

Authors’ additions based on review of the questionnaires across the three survey programs. The achieved sample size refers to the final sample after harmonization across surveys (see text for details). Where information was unclear or inconsistent, we contacted the survey programs directly for more details

The DHS surveys are nationally representative, and issue multi-stage stratified random samples where enumeration areas are randomly selected with a probability proportional to their population. Households are then randomly selected within each enumeration area. The FTF surveys are representative of the population in the FTF zone of influence (typically multiple first-level administrative divisions) and use multi-stage random sampling, except in a few countries (here, Cambodia and Mozambique) where samples are not stratified. In Bangladesh, the FTF survey was only conducted in rural areas within the geographical areas of focus. To make the populations as comparable as possible, we limit the DHS samples to areas covered by the FTF survey in each country.

Once households are identified, the two survey programs use different strategies to identify primary respondents within the household. FTF interviews the lead female decision maker, usually the wife of the main male decision maker or household head. In most countries, the DHS interviews all women aged 15–49 in target households; in select countries (here Bangladesh), only ever-married women are interviewed. To ensure comparability across the survey programs, we limit our sample to dual-headed households^3^ and to women who are either the wife of the male household head or the household head themselves. Although our approach means that the estimates presented in this paper are not nationally representative, it does allow us to compare similar groups so that we can isolate differences attributable to question design or survey features. Details on similar harmonization for the LSMS-ISA survey sample are in the Online Resource. Table 1 reports the effective “post-harmonization” sample size for our analysis that retains only dual-headed households in the DHS and limits it to the same geographies as the FTF. Table A.2 provides information on languages used and the approach to translation, while Tables A.3–A.6 present descriptives on key individual and household characteristics. As expected, given the focus on women of reproductive age in the DHS, the women in the FTF and LSMS are generally 3–7 years older than those in the DHS. Other differences across survey programs vary considerably by characteristic and country, with no clear patterns emerging.

### Outcome Indicators

We harmonize responses to survey questions on control over income and decisionmaking on large household purchases across the three survey programs (Table 2). Responses to the control over income indicator were classified into two categories, “no input or control” and “some/all input or control.” For each activity asked about in the FTF, responses of no input and input into few decisions were dichotomized as “no input or control”; all other responses were classified as “some/all input or control”.^4^ If a respondent did not participate in an activity (a response of “no decisions made”), they are coded as not having control over income from that activity.^5^ The overall indicator aggregates these activity-wise responses, such that a respondent who was recorded as having some/all input or control in any one activity was classified as such for the overall indicator.

**Table 2:** Full text of questions, respondents and responses used to create indicators in FTF and DHS

FTF		DHS	
Question/response options	Respondent	Question/response options	Respondent
*Measure: Control over income from any source*			
*[If participated in the activity in the past 12 months]* How much input did you have in decisions on the use of income generated from [ACTIVITY]?; where [ACTIVITY] includes food crop farming, cash crop farming, livestock raising, non-farm economic activities, wage and salary employment, fishing or fishpond culture Response options: No input or input in few decisions; Input into some decisions; Input into most or all decisions; No decisions made	Primary female respondent, usually the wife of the main male decision maker or household head	*[If female respondent has done any work in the last 12 months]* A. Who usually decides how the money you earn will be used? *[If female respondent’s spouse has done any work in the last 12 months]* B. Who usually decides how your husband’s/partner’s earnings will be used? Response options: Respondent; Husband/partner; Respondent and husband/partner jointly; Husband/partner has no earnings (only for B); Other	Women aged 15–49 in target households*
**Approach to harmonization:** Converted into two response categories: no input or control and some/all input or control. For FTF, responses of no input or input into few decisions and no decisions made were grouped into "no input or control:" all other responses were classified as "some/all input or control." For DHS: respondent was classified as having "no input or control" if she did not participate in the decision at all, and as "some/all input or control" if they were named as decision-maker either alone or with someone else. In the FTF, if the respondent indicated some/all input or control over any one component, she was classified as having some/all input or control overall			
*Measure: Decisionmaking around large HH purchases*			
When decisions are made regarding major household expenditures, who is it that normally takes the decision? Response options: Self; Spouse; Other HH member; Other non-HH member	Primary female respondent, usually the wife of the main male decision maker or household head	Who usually makes decisions about making major household purchases? Response options: Respondent; Husband/partner; Respondent and husband/partner jointly; Other	Women aged 15–49 in target households*
**Approach to harmonization:** Converted into two response categories: did not take the decision and took the decision solely or jointly. For both FTF and DHS the respondent was classified as having no input if they did not mention self, and as having some/all input if they mentioned self alone or self with another individual			

^*^In Bangladesh, only ever-married women are interviewed for the DHS

For DHS, the questions were phrased as “Who usually decides how the money you earn will be used?” (missing if the respondent did not earn an income) and “Who usually decides how your husband/partner’s earnings will be used?”. The respondent was classified as having “no input or control” if she did not participate in these decisions for either source of income, and as “some/all input or control” if she listed herself as decision-maker either alone or with someone else for at least one source.

The indicator for decisionmaking on household purchases was also classified into two response categories: did not take the decision and took the decision solely or jointly. For both the FTF and DHS the respondent was classified as not taking the decision if they did not mention themselves as one of the decisionmakers, and as taking the decision solely or jointly if they mentioned themselves as a decisionmaker, either alone or along with another individual. Because perceptions of sole and joint decisionmaking can differ across contexts (Seymour & Peterman, 2018), we avoid assuming a hierarchical power structure between sole and joint decisionmaking and instead combine them into a single category.

A description of the construction of outcome indicators for the LSMS-ISA survey program is provided in the Online Resource, with the full text of the questions reproduced in Table A.7.

## Descriptive Statistics

**Fig. 1:**
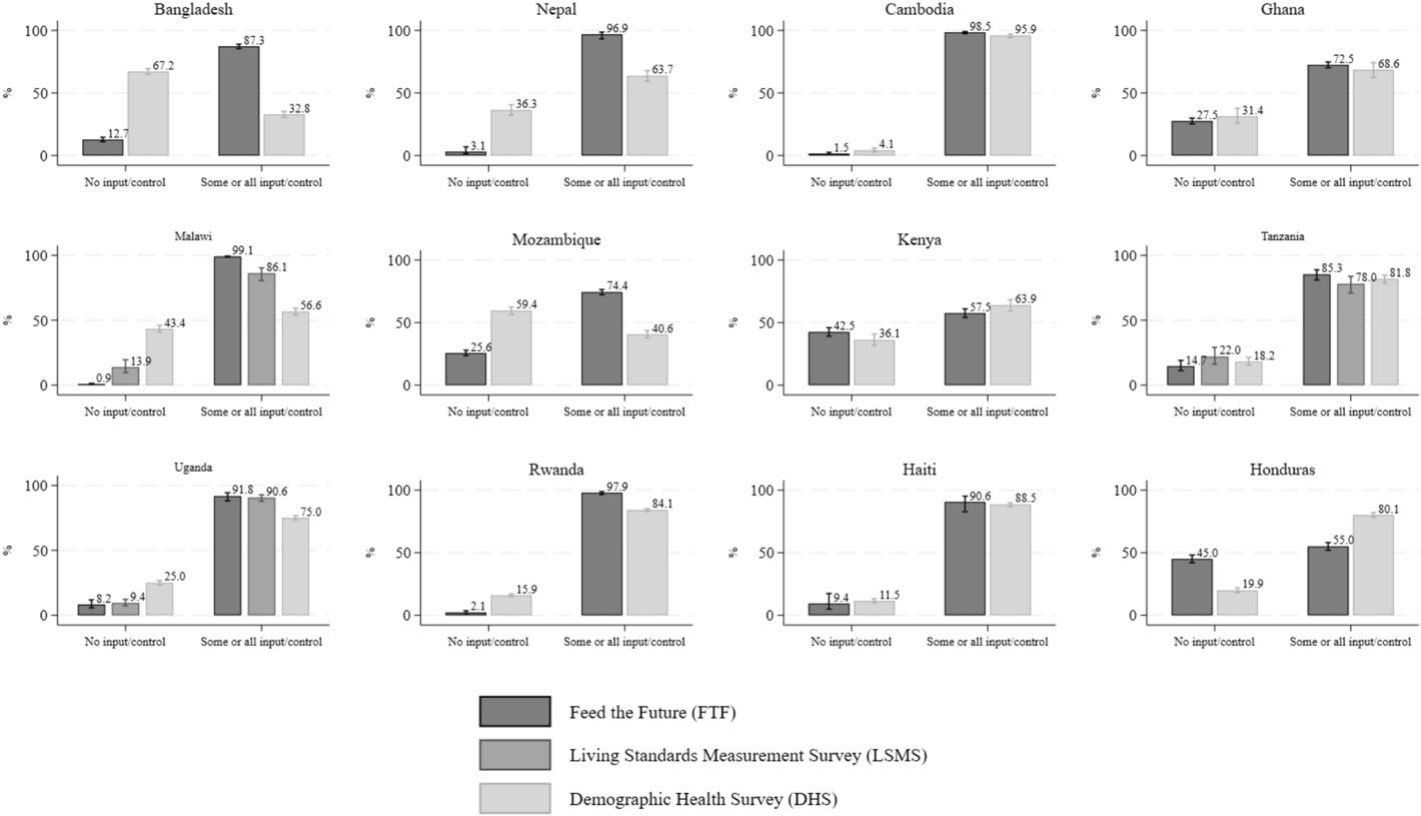
Comparison of women’s control over use of income by survey program

Figure 1 presents the raw descriptives for control over income, with 95% confidence intervals. For Cambodia, Ghana, Kenya, Tanzania and Haiti, the FTF and DHS estimates of these proportions are roughly in line with one another but could still lead to divergent conclusions. In Bangladesh, Nepal, Malawi, Mozambique, Uganda, Rwanda and Honduras, FTF and DHS give statistically significantly different estimates of the proportion of women decision makers that have some or all control over use of income from any source. Except for Honduras, where 55% of FTF and 80.1% of DHS decision makers are classified as having some or all input or control over use of income, when different, the FTF estimate of control over use of income is generally larger than the DHS estimate, starkly so in Bangladesh, Nepal and Mozambique.

LSMS-ISA is only available for three countries in our sample. In Malawi and Uganda, the differences between the LSMS-ISA and the DHS are statistically significant, with LSMS-ISA overestimating control over income relative to DHS but underestimating it relative to FTF. In Tanzania, the LSMS-ISA underestimates control over income relative to the other two surveys, but not significantly: 78% in the LSMS-ISA, 81.8% in the DHS and 85.3% in the FTF are recorded as having some or all control over use of income.

It is possible that these differences reflect modest improvements across years. In the case of Tanzania, this is plausible, since the LSMS-ISA, DHS, and FTF occurred in that order approximately a year apart from one another, with improvements of around 4 percentage points (pp) between each survey or year. However, this hypothesis does not hold for either Malawi or Uganda. In Malawi, the DHS took place a little over two years after the FTF, and LSMS-ISA began another 14 months after DHS. In Uganda, the LSMS-ISA and FTF surveys started in the same month—with FTF lasting 3 months and LSMS-ISA lasting a year—and do lead to similar estimates, but the DHS survey began mere months after the LSMS-ISA ended and its estimates of women’s control over income are approximately 15 pp lower than those of the LSMS-ISA.

**Fig. 2:**
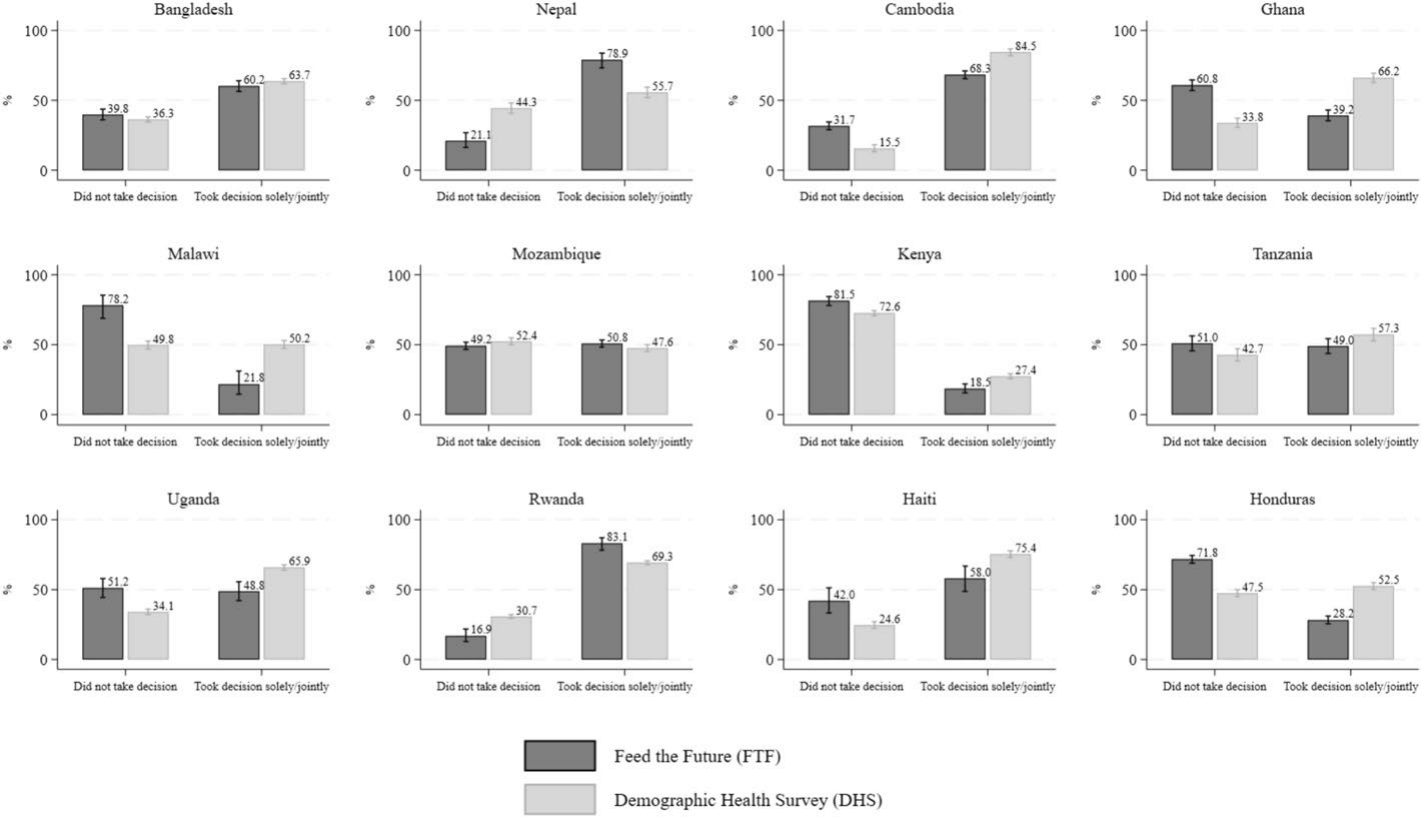
Comparison of women’s input into large household purchases by survey program

Figure 2 presents similar results for the second outcome measure of input into decisions over large household purchases. With some exceptions, notably Bangladesh, Mozambique, and Tanzania, estimates differ quite substantially across survey programs despite sample harmonization, with non-overlapping 95% confidence intervals. In Nepal and Rwanda, the proportion of women decision makers reported as solely or jointly making decisions over large household purchases is statistically significantly larger in the FTF than in the DHS (78.9% in FTF vs. 55.7% in DHS in Nepal; 83.1 in FTF vs. 69.3% in DHS in Rwanda). In the remaining countries this pattern is reversed, with an FTF estimate of sole or joint decisionmaking power lower than the DHS estimate by 10–30 pp.

The differences across survey programs within a country in control over income and decisionmaking over large household purchases could also reflect differences in how questions are framed in the two surveys. To check if these differences persist also for other variables not related to women’s empowerment, we present similar graphs for two arguably more “objective” measures—whether the household has a bicycle and whether it has access to electricity (Figs. A.1 and A.2 in the Online Resource). It is clear from these figures that when we use these arguably less complicated and more objective measures unrelated to women’s empowerment, differences across surveys within countries are small in both magnitude and number. For bicycle ownership, the differences are not statistically significantly different for any country for which this information is available (Fig. A.1). For electricity access, we do see some differences—in Honduras, Ghana and Cambodia—but in all other countries, the confidence intervals for the proportions reporting having access to electricity in the DHS and FTF overlap (Fig. A.2).

What could explain the differences across survey programs in estimates of the proportion of primary females that have control over income or exercise decisionmaking power? Next, we present possible hypotheses and discuss how we control for these sources of bias in the regression results that follow.

## Hypotheses for Variation in Measures of Women’s Empowerment Across Survey Programs

Underlying our exercise in comparing responses across surveys is the assumption that each survey aims to measure the same construct and that differences in design can affect how closely the survey approximates underlying values. In the case of decisionmaking and control over use of income, the underlying construct is unobservable, so we can never know which measure is “more accurate.” This is in contrast to other outcomes like time-use, plot size or crop yield, subject to extensive treatment in the measurement error literature, where there exist “gold standard” measures.

Potential causes of deviation across surveys are variation in who responds to the question and in how the question is posed, the respondent’s comprehension of the question, their perception of and ability to measure the underlying construct or their incentives to misrepresent it, or social desirability bias. Some of these may be attributed to survey design and implementation; others, such as measurement error or social desirability bias, could be attributed at least in part to the characteristics of the individual respondents.

We draw on the existing literature on survey design to present hypotheses for the variation in survey responses even when assessing the same outcome among similar populations. We divide these hypotheses into those related to survey *structure*, i.e., survey length, the language of the translation, or the way questions are framed, and those related to survey *administration*, i.e., the relationship of the enumerator to the study population, the presence of other members of the household at the time of interview, the languages in which the interviews were conducted and so on. Depending on the hypothesis and its testability, we describe how we use our data to test or account for possible biases.

### Survey Structure

#### Survey Length

Long survey instruments raise concerns of respondent burden (Rolstad et al., 2011) and data quality (Choi et al., 2014), especially for questions that occur later in the interview. Inaccurate responses could arise from respondent fatigue and consequent inability to provide careful responses, or from a deliberate attempt by respondents to shorten a long interview, say by repeated acquiescing (a process known as “satisficing” (Krosnick, 1991)) or responding “no” in order to skip anticipated follow-up questions. Sometimes these responses might even be encouraged by a tired interviewer who is familiar with the skip patterns and wants to shorten the interview. Short and long versions of survey questionnaires administered to the same households can yield different answers, though the exact mechanisms for this are not clear (Kilic & Sohnesen, 2019). In their study of the relationship between length and data quality in DHS surveys in four countries in sub-Saharan Africa, Choi et al. (2014) find that longer individual interviews were associated with greater inconsistencies in reporting. Three recent papers find significant and sometimes large effects of survey fatigue. In an experiment in rural Ghana, Ambler et al. (2021a, b) randomize the order in which household members appear in the labor module and find that those appearing later are reported as participating in fewer work activities than those listed earlier. Abay et al. (2021) find that when a dietary diversity module is administered later in a long survey, there is a significant reduction in the reporting of foods eaten, especially those that are infrequently consumed, even though this specific module did not trigger a skip pattern for which more details were requested. Finally, Jeong et al. (2022) randomize the order of questions across multiple rounds of panel surveys to show that respondents are more likely to skip questions that appear later in the survey, with large impacts on measures of food and other expenditures.

Our analysis relies on existing secondary datasets, so we cannot systematically vary the length or randomize the order in which modules appeared. Each of the surveys we use covers multiple topics and is subsequently quite lengthy. In the DHS, questions on both control over income and decisionmaking on large household purchases are asked toward the end of women’s interviews (page 64 of 75 in the standard Phase 7 questionnaire, page 48 of 59 in the standard Phase 6 questionnaire). In the FTF surveys, there is more inter-country variation. In some, like Bangladesh, Cambodia, Ghana, Honduras, Kenya and Uganda, the WEAI is embedded in a lengthy questionnaire, following detailed consumption or agriculture modules. In others, like Haiti, Malawi, Nepal, Rwanda and Tanzania, the overall questionnaires were much shorter and the WEAI is the main component. Table A.2 in the Online Resource describes the placement of the relevant module within the larger household surveys.

In the FTF surveys, the question about control over income is the third in a set of three questions asked about each activity—the first being “Did you yourself participate in [ACTIVITY] in the last 12 months?” and the second “How much input did you have in making decisions about [ACTIVITY]?.” A “No” to the first question skips the second and third questions, while a “No decision made” to the second results in skipping the third. Since this set of three questions is repeated for six activities, a respondent wishing to speed up a lengthy interview could conceivably alter their responses in a way that would also lead to a lower proportion of FTF respondents reporting control over income from income sources. Note that this is not what the descriptives above suggest. In contrast, the DHS asks the questions on control over income directly, with no accompanying skip patterns that a respondent could “game” to reduce interview times.

Similarly, the question on input into decisions about household expenditures in the FTF is part of a survey module asking about seven activities (getting inputs for agricultural production, livestock raising, etc.) of which major household expenditures is the sixth item. The survey module includes a follow-up question about whether the respondent feels they would be able to make their own personal decisions about the activity should they choose, which is triggered if the respondent reports jointly making decisions in the first question. A respondent trying to reduce the length of the tool could either report sole decision-making or state that the activity was not applicable to them. Both actions result in the follow-up question being skipped, but have different implications on the indicator itself, making the direction of bias unclear. As with control over income, the DHS question structure is comparatively simple, with no skip patterns that can be easily gamed to reduce length.

#### Language and Translation

Interview language has a direct bearing on the respondent’s ability to understand and accurately respond to surveys. Both survey programs we analyze typically translate tools into the primary spoken languages of the country in question (see Table A.1 for details); for less commonly spoken languages translations can sometimes occur ‘on the fly,’ or respondents may be forced to reply in a language in which they are less fluent. Ad-hoc translations can introduce disparities across enumerators for a single question and within enumerators across respondents. Depending on the depth of training, different enumerators may themselves understand the questions differently. A limited understanding of the question could manifest in non-response, or alternatively, in satisficing, which could either inflate or deflate reports of decisionmaking. Overall, it is not clear that these errors would lead to bias in a specific direction, as opposed to just more noise. Weinreb and Sana (2009) show using the Kenyan DHS that non-standardized ‘on the fly’ translations were associated with greater interviewer-related variance but had only a small effect on response values; however, these seemingly small differences were magnified in more complex multivariate forms of analysis. We expect that translation issues could result in larger errors in reporting on the kinds of outcome measures used in this paper, which involve nuances around involvement and input in decisions. Unfortunately, the FTF and DHS are not consistent in recording whether the language of the survey matched the respondent’s primary language: the DHS only has this information for Malawi and Uganda, and the FTF only records it for Cambodia, Malawi, Nepal, Rwanda and Uganda.

Our hypotheses for this aspect of survey design are two-fold. Building on Weinreb and Sana (2009), we do not expect to see large discrepancies in the levels of our outcome measures, even in countries where a larger proportion of respondents are interviewed in a language that is not their primary language. However, we do expect such issues to cause an attenuation in the association of our measures of empowerment with individual and household-level characteristics. Table A.1 suggests that DHS provided the broadest range of translated tools (except in the case of Cambodia).

#### Cognitive Difficulty or Framing

The complexity of the question or response format can affect accurate responses and overall estimates, along the same lines as the arguments above on language comprehension (Willis & Miller 2011). The FTF and DHS differ considerably in their phrasing of questions around control over income (see Table 2 and the discussion in 4.1.1 above). The FTF asks “How much input did you have in decisions on the use of income generated from [ACTIVITY]?” with responses in terms of the extent of input, requiring enumerators to exercise judgement or probe further if the respondent provides an answer that is not phrased in the same terms. In fact, results from later cognitive interviewing on similarly structured questions suggest reordering the phrases to first reference the activity before asking about decisions related to the activity (Hannan et al., 2020). In contrast, the DHS presents these questions in a similarly straightforward manner, “Who usually decides how the money you earn will be used?” A priori, as with the language comprehension questions, we might expect an attenuation of the association of our outcome measures with covariates for surveys where the complexity of the question increases response variance, i.e., weaker associations for the FTF than for the DHS.

For decisionmaking, the FTF phrasing—“When decisions are made regarding major household expenditures, who is it that normally takes the decision?”—and the DHS phrasing—“Who usually makes decisions about making major household purchases?” are very similar, as are the response options. We do not expect that differences in cognitive difficulty of the two questions drive significant differences across these two survey programs.

### Survey Administration

In addition to aspects of survey structure discussed above, surveys with the same questions and the same structures can elicit different responses depending on characteristics of survey administration. These include respondent selection, whether the respondent is interviewed in the presence of others or alone, and when the survey is conducted.

#### Choice of Respondent

When measuring decisionmaking, it matters who is being asked. Having household members respond for themselves is widely considered to be best practice (De Weerdt et al., 2020), and studies have noted discrepancies in reported decisionmaking between partners (Annan et al., 2019; Seymour & Peterman, 2018). Soliciting responses through proxy (i.e., another household member answering on behalf of the target respondent) has been shown to lead to underreporting of employment and of women’s ownership over assets by male members of the household (Kilic et al., 2020, 2021). A priori, we might expect that proxy reporting would bias downward the proportion of women reported as having control over income or participating in decisionmaking.

In this paper, proxy reporting is less of a concern as women respond about their own input into decisionmaking and control over income in both the FTF and DHS (Table 2). This is not the case in the LSMS-ISA, where these questions could be posed to the household head or most knowledgeable member, who is asked to list up to two household member IDs (Table A.7). Unfortunately, since the control over income questions in LSMS-ISA are obtained from multiple modules for whom the identity of respondent is not reported, it is not straightforward to estimate how often women are reporting for themselves.

Another aspect of the choice of respondent that might affect survey responses is the real or perceived relationship between the respondent and the enumerator. Social distance from the enumerator, as manifest in shared or distinct social, ethnic or religious identities, could influence responses in different ways (see, for example, Adida et al. (2016) and Cilliers et al. (2015) for evidence on the role of shared interviewer-participant ethnicities from Africa, and Blaydes and Gillum (2013) for interesting results on the impact of religiosity of the interviewers on survey responses in Egypt). Di Maio and Fiala (2020) and Fiala and Masselus (2022) suggest, however, that enumerator effects are likely to be limited, except in the case of sensitive questions. Our hypothesis that social distance might bias reports of women’s control over income or decisionmaking does not have a clear direction, but we can control for these aspects of social distance and for differences in enumerator characteristics by including both enumerator fixed effects and basic respondent demographics into our analyses (see Sect. 5 below).

#### Presence of Others

The presence of someone else in the household or at the interview, a spouse or an in-law, might encourage the respondent to report ‘model’ behavior or to hide information. Ambler et al. (2020) note that women in households with in-laws underreport participation in decisionmaking and the ownership of assets, and that overall concordance between husband and wife about the wife’s role and ownership is higher when in-laws are not present. Fiala and Masselus (2022) show that survey responses vary depending on whether spouses are interviewed together or separately, though the differences aren’t systematic. On the sensitive topic of wife beating in Pakistan, Raza and Pals (2024) show that women whose interviews are overheard respond more positively towards wife-beating. To the best of our knowledge, however, there is no evidence of the direction in which the presence of others might bias own- or proxy-reported control over income or participation in decisionmaking.

In our sample, we note sizeable differences across surveys in the proportion of respondents who were interviewed alone in Cambodia (FTF 17%, DHS 99%), Mozambique (FTF 85%, DHS 95%), Tanzania (FTF 76%, DHS 99%) and Uganda (FTF 97%, DHS 89%) (Table 3). With the exception of Cambodia, where the proportion of respondents interviewed alone for the FTF was very small, more than 75% of the respondents in each country-survey combination were interviewed in private, reducing concerns that this factor is key to driving variation across surveys within a country.

**Table 3:** Proportion of respondents who were interviewed alone by survey and country

Country	FTF (%)	DHS (%)
Bangladesh	95.2	< *not reported* >
Cambodia	16.7	99.3
Ghana	96.4	< *not reported* >
Haiti	98.3	92.8
Honduras	92.0	89.1
Kenya	92.2	94.4
Malawi	89.2	93.2
Mozambique	85.4	95.2
Nepal	98.5	96.7
Rwanda	99.1	97.6
Tanzania	76.4	99.4
Uganda	97.0	88.7

Authors’ calculations from datasets. For the DHS surveys, the variable used is whether the respondent was interviewed alone for the domestic violence module and hence may be a noisy measure of being interviewed alone for the whole survey

#### Seasonality and Survey Timing

The last aspect of survey administration that could affect responses to survey questions is the timing of the survey, especially for questions that have a seasonal dimension, for example, decisionmaking on agriculture and the use of income from seasonal activities, both farm and off-farm. Reports of income from crop sales, for example, could be more strongly impacted by recall error the further data collection is from harvest and sale. Seasonality has been shown to affect responses to food frequency questionnaires (Fowke et al., 2004) and employment (Comblon & Robilliard, 2015), though less is known about its impact on reported control over income or decisionmaking. This issue is made more complex by the range of countries with different agricultural calendars, and differences across FTF, DHS and LSMS survey months within a country (Table 1). We account for this by including month fixed effects in our analysis; see Sect. 5 below for details.

### Summary of Hypotheses

Table 4 summarizes our hypotheses regarding the role of survey structure and administration in introducing variation in responses, the expected direction of bias, if known, and the approach taken to account for this in this paper.

**Table 4:** Summary of hypotheses regarding possible variation in responses across surveys within a given country

Survey characteristic	Description	Summary of hypotheses	Approach to addressing these differences within this paper
*Survey structure*			
Survey length	All three surveys are long. The placement of the modules from which the control over income and decisionmaking questions are drawn varies across countries, but in all cases is far enough in to where respondents are experiencing survey fatigue. In the FTF and LSMS-ISA surveys, there are more opportunities for learning from previous, similar questions, which may lead respondents or enumerators to “game the system”—but this could lead to either under- or over-reporting of input	Given placement, don’t expect this to be a significant cause of variation across surveys within a given country	None
Language and translation	In general, the DHS surveys appear to have done the most to ensure that survey instruments were translated into local languages, and that these translations were incorporated into the data collection programs and not left to enumerators to perform "on the fly."	We expect greater attenuation of associations between measures of empowerment and individual and household characteristics where there is greater language mismatch	Control for a mismatch between the primary language of the respondent and the language in which the interview was conducted, where this information is available
Cognitive difficulty	For control over use of income, FTF surveys have the most complex framing of question and response options, but it is not obvious how this might bias responses. FTF and LSMS-ISA questionnaires are designed such that a respondent wishing to reduce time on the survey could underreport. In comparison, DHS questions and response options for control over use of income questions are relatively straightforward. Questions on decisionmaking on large household purchases are phrased similarly in the FTF and DHS, as are the response options	We expect greater measurement/reporting error and hence greater attenuation of associations between measures of empowerment and individual and household characteristics where the questions are more complex	None
*Survey administration*			
Choice of respondent and social distance from enumerator	Both FTF and DHS address questions to the primary female respondent in question. The LSMS-ISA uses proxy reporting, addressing these questions instead to the head of the household or the most knowledgeable household member. It also allows for the report of up to 2 household members only	Unclear, given limited literature on impacts on decisionmaking questions. We might expect proxy reporting to bias downward the proportion of women reported as having control over income or participating in decisionmaking	Including both enumerator fixed effects and basic respondent characteristic accounts for social distance (though not for proxy reporting)
Presence of others at the interview	Could affect reports of control over use of income and decisionmaking downward if respondent woman wants to conceal this information from in-laws or other relatives	Don’t expect significant discrepancies across surveys within a country given that the proportion being interviewed in private was generally high. Cambodia is a notable exception to this	None needed
Survey timing	Could affect reports of control over use of income and decisionmaking over activities that are highly seasonal, e.g., crop sales, livestock sales etc. If asked in the off-season, recall bias might attenuate reports	Expect greater measurement error in surveys conducted in the off season; could be counterbalanced by limited cognitive capacity of the respondent in busy periods of work. Overall, unclear	Control for month FE in the regression analysis to account for varying crop calendars and survey months across countries

The reasons for divergence between surveys that we mention above are not unique to surveys of women’s control over use of income or their decisionmaking but apply more generally to any kind of survey. However, we do expect that some of these are more relevant for our outcomes of interest. In terms of survey structure, language and translation and cognitive difficulty are likely to matter greatly; in some languages there is no clear equivalent for “empowerment,” “control” or “decisionmaking,” and even where there is, questions are understood differently or only responded to partially (Hannan et al., 2020; Myers et al., 2023). Aspects of survey administration could also be more salient for the measurement of control over use of income and decisionmaking than for other types of outcomes, for example, reported control over use of income might vary by when that income is earned and can be spent, or by whether the mother-in-law is listening to the conversation.

## Regression Analyses

### Methods

Section 3 showed the substantial differences between the levels of our outcome measures in the FTF and DHS, despite our attempts at harmonizing the sample and the response categories. For control over income measures, there are five countries where FTF and DHS measures are comparable. Among those where differences are large and statistically significant, it was generally the case that FTF overestimated control over income relative to DHS. Where this data was available, the LSMS-ISA typically overestimates control over income relative to the DHS but underestimates it relative to the FTF. For decisionmaking, we still see substantial differences across FTF and DHS estimates, despite the similarity in wording of the question and the response options. FTF underestimates decisionmaking relative to DHS in 7 of the 12 countries, overestimates it in two, and is comparable in three.

In this section, we describe the multivariate ordinary least squares (OLS) regressions we use to estimate associations between our outcome measures and commonly used individual and household level characteristics (age and education of the primary female, household size and wealth quintiles) as well as subnational dummies using the appropriate geographical unit. A priori*,* we would expect that both age and education would be positively correlated with improved measures of women’s empowerment. Household size could go either way—larger households mean more decisions to be made, but also more people who could potentially make those decisions. Household wealth too is ambiguous. Following the discussion in Sect. 3, we include interviewer and month fixed effects and a dummy for language mismatch to help account for the survey structure and administration characteristics presented in Table 4.

Specifically, for a woman $i$ in household $h$ in subnational area $s$ in survey-month $m$ interviewed by enumerator $j$ we estimate the following using ordinary least squares separately for each country and survey combination:

1 $$Y_{ihsmj} = \alpha + \beta_{1} X_{ihsmj} + \beta_{2} Z_{hsmj} + \gamma_{s} + \mu_{m} + \delta_{j} + \varepsilon_{ihsmj}$$

where ${Y}_{ihsmj}$ is the binary individual-level outcome of interest, control over income or decisionmaking over large household purchases, with no input/control (for control over income) and did not take decision (for decisionmaking) coded as 0.

${X}_{ihsmj}$ is a vector of individual-level characteristics, including the current age of the primary female, her education (dummy for primary education or greater, base category: incomplete primary or no schooling), and (where available) a dummy for a mismatch between the language of the survey questionnaire and the respondent’s native language.

${Z}_{hsm}$ is a vector of household characteristics, specifically household size and six wealth quantile dummies (base category: poorest wealth quantile). Wealth quantiles were generated using the International Wealth Index approach (Smits & Steendijk, 2015).^6^

${\gamma }_{s}$ are subnational fixed effects, based on survey structure (Table 1).

${\mu }_{m}$ are month fixed effects.

${\delta }_{j}$ are interviewer fixed effects (where available).

The vectors of coefficients, ${\beta }_{1}$ and ${\beta }_{2}$, and the individual-specific error term, ${\epsilon }_{ihsmj}$ are to be estimated.

### Differential Associations Between Individual Characteristics and Women’s Empowerment Across Surveys

**Fig. 3:**
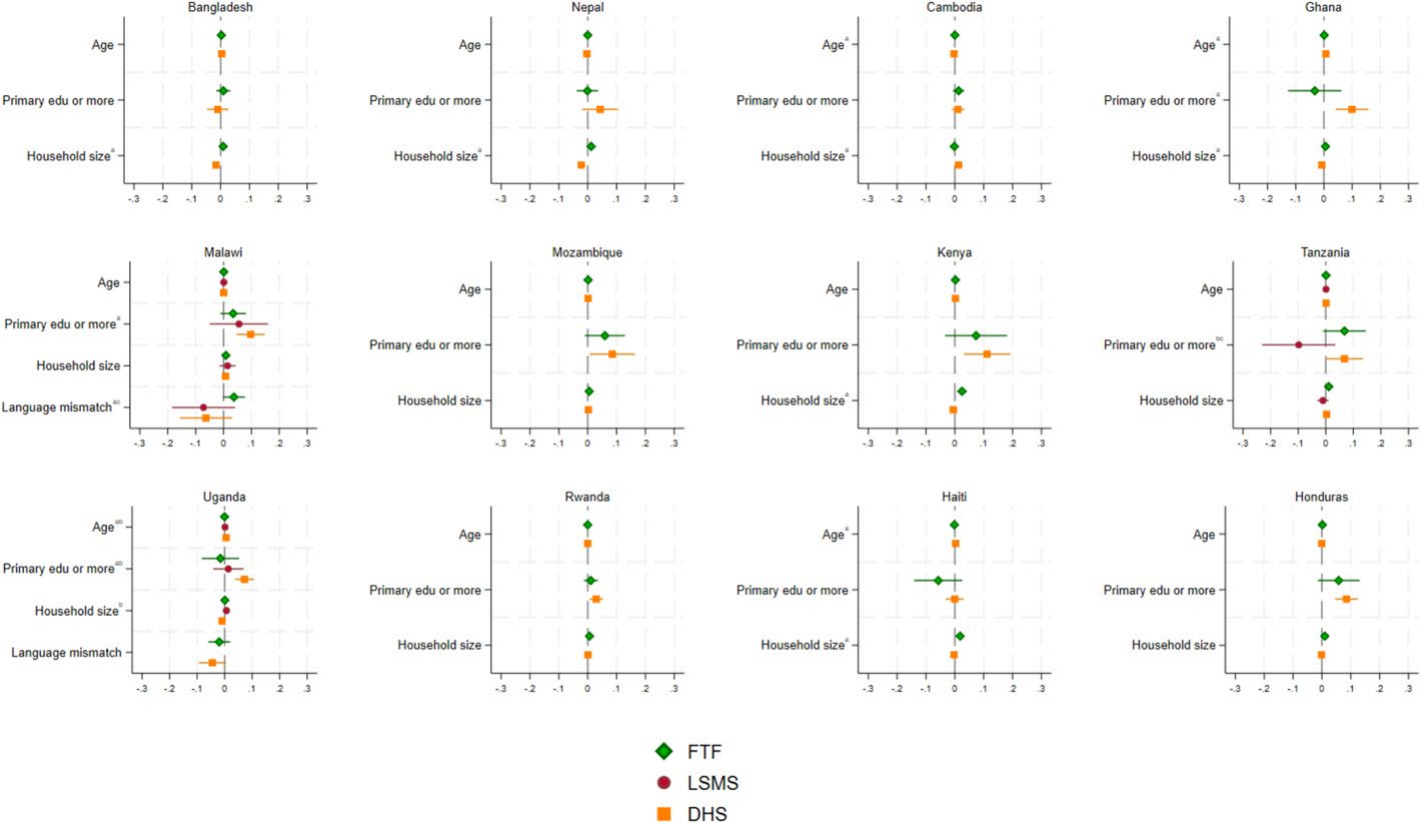
Characteristics associated with women’s control over use of income by survey program in 12 countries. A note on superscripts: **a**: differences between FTF and DHS are statistically significant, **b**: differences between DHS and LSMS are statistically significant, **c**: differences between FTF and LSMS are statistically significant. Estimates are also reported in the Online Resource

**Fig. 4:**
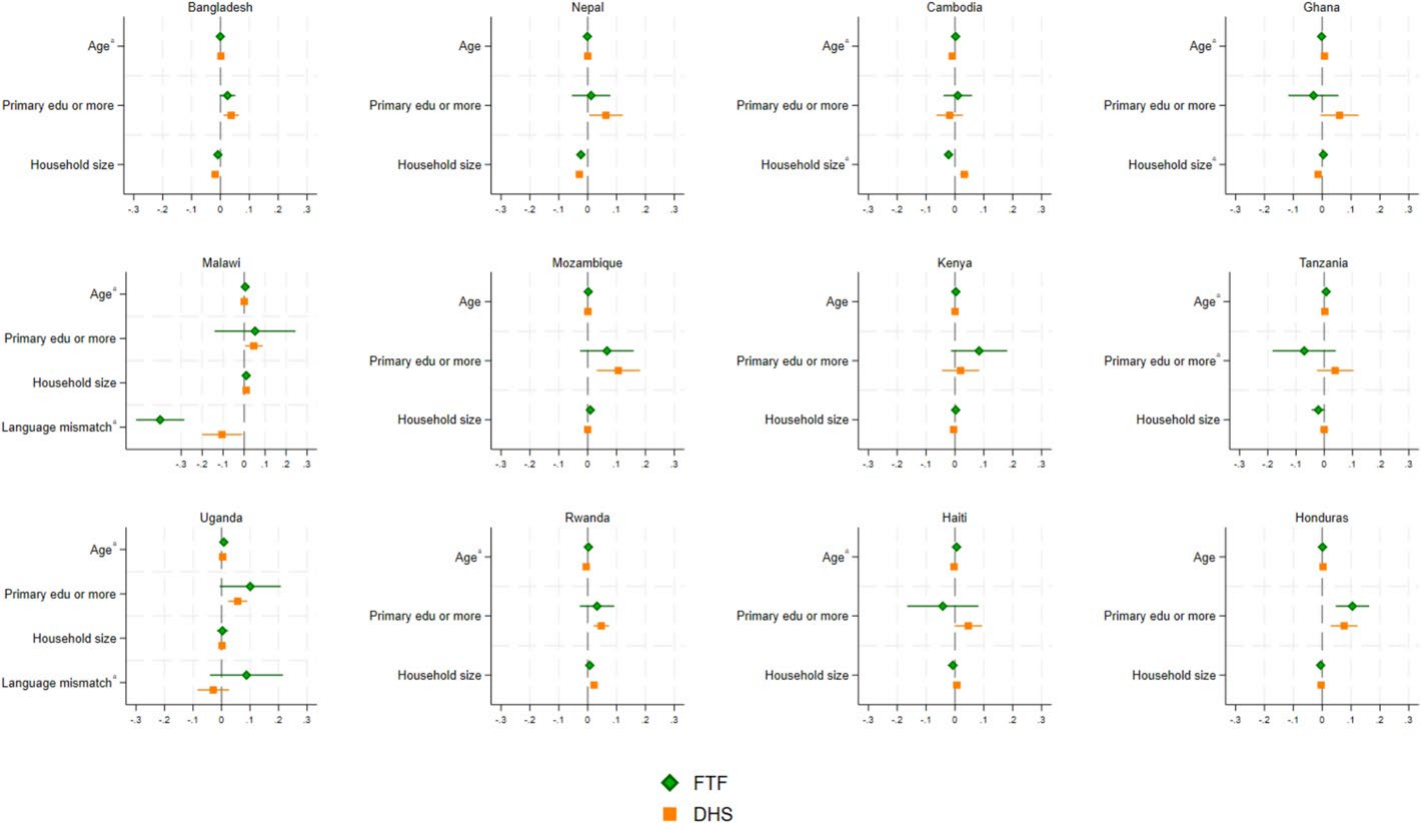
Characteristics associated with women’s decisionmaking by survey program in 14 countries. A note on superscripts: **a**: differences between FTF and DHS are statistically significant, **b**: differences between DHS and LSMS are statistically significant, **c**: differences between FTF and LSMS are statistically significant. Estimates are also reported in the Online Resource

The associations of our measures of women’s empowerment with the individual and household characteristics described in Eq. (1) are graphically depicted in Figs. 3 and 4. Coefficients on the wealth quantile variables were mostly statistically insignificant from zero and have been omitted. The full regression results are in the Online Resource.

For control over use of income (Fig. 3), associations with age, education and household size are roughly the same across survey programs for Bangladesh, Nepal, Cambodia, Mozambique, Kenya, Rwanda, Haiti and Honduras. In these cases, even where the point estimates are statistically significantly different from each other, they are not indistinguishable from zero (e.g., household size in Bangladesh, Nepal, Cambodia, Kenya, or age in Haiti). In other countries, we do see differences across survey programs. In Ghana, for example, we see a statistically significant difference in the coefficient on education, with the FTF point estimate considerably smaller (and negative), compared to the statistically significant and positive education coefficient for the DHS. We see a similar pattern on the association of education and control over income in Malawi, Uganda and Honduras, though the difference is not always statistically significant. As a reminder, our hypotheses around language mismatch and complexity of the question both suggested greater attenuation in the FTF multivariate associations.

In Malawi, Tanzania and Uganda, where estimates from the LSMS-ISA are also available, the bulk of the statistically significant differences are in associations with primary education. Here the LSMS-ISA point estimates lie between the FTF and the DHS for Malawi and Uganda, which is in line with our discussion in Sect. 3 on the complexity of the question and response options.

For women’s decisionmaking (Fig. 4), we see minor discrepancies across survey programs in the associations between our outcome and key covariates, and most of these are in the association with age (in years), where the point estimates are small (the case for Bangladesh, Cambodia, Ghana, Malawi, Tanzania, Uganda, Rwanda, Haiti, Honduras). Other differences in potential determinants across survey programs are largely statistically insignificant, as one might expect given that both the FTF and DHS use very similar language to phrase these questions and response options, and that both translate their surveys into a comparable number of languages (see Sect. 4 and Table A.1). We do see larger differences in the case of education, where the point estimates are larger and more statistically significant for the DHS relative to the FTF in the case of Bangladesh, Nepal, Ghana, Mozambique, Tanzania, and Haiti.

## Discussion

In this paper we use data from two international survey programs—FTF and the DHS—and 12 countries in Asia, Africa, Central America, and the Caribbean to construct comparable indicators of women’s control over income from any source and of decisionmaking, and to assess within-country inter-survey differences in both the levels of these measures and their associations with commonly used individual- and household-level characteristics. Although the two survey programs focus on different themes (agriculture and food security for FTF, and population, health, and nutrition for DHS), both view women’s empowerment as important potential drivers of these outcomes and have gone to considerable lengths to develop and include women’s empowerment measures. For the three countries in our sample where LSMS-ISA data was also available, namely Malawi, Tanzania and Uganda, we also compare the levels and associations for that survey program.

After harmonizing geography, choice of respondent, survey timing and the construction of outcome measures to ensure maximum possible comparability, we find that large differences persist in the percentage of women considered empowered according to each indicator. We summarize key hypotheses for these differences, related to survey structure and survey administration. We discuss the direction in which we expect these survey characteristics to affect multivariate analyses of associations between our outcome measures and individual- and household-level characteristics and outline how we can begin to account for some of these discrepancies. We then estimate multivariate models using additional controls for various forms of inter-survey variation. We find some statistically significant differences in the associations of control over income with women’s education (for example, in Ghana, Malawi and Uganda) and with household size (for example, in Bangladesh, Nepal, Cambodia, Ghana, Kenya and Haiti) but these generally accord with our a priori expectations given the survey characteristics that could not be accounted for in the analysis. Given how differently the decisionmaking questions were posed in the FTF and DHS, it is surprising that we do not see more statistically significant differences in the association of the measure of decisionmaking with key covariates, other than associations with respondent women’s age in the case of Bangladesh, Cambodia, Ghana, Malawi, Tanzania, Uganda, Rwanda and Haiti.

There are several limitations to our analysis, providing clear direction for future research. First, unlike several of the papers reviewed in Sect. 4, we were limited in our ability to directly test our hypotheses on aspects of survey structure and administration because we used secondary data. This meant that key variables, such as module-wise time stamps, now a regular feature of tablet-assisted data collection, were not available, and we could not randomize aspects of the survey, such as module placement or the use of the respondent’s primary language. Addressing these questions with survey experiments will help us understand the specific consequences of survey design characteristics.

A second limitation is that in attempting to harmonize surveys across programs, we were forced to alter the geographic scope and restrict our respondents to women living with their husbands. This limits the representativeness of the data and potentially the generalizability of the findings. These data sources also restricted our ability to compare associations between women’s empowerment and a broader range of characteristics and outcomes that may be associated with women’s empowerment.

A third limitation is that given the large number of languages into which survey tools were translated (either formally or on-the-fly) and the absence of the translated versions of the tools, we were unable to fully account for the cognitive difficulty of the wording. Going forward, it might be possible to use advanced text analysis methods to quantify the cognitive complexities.

Our work can be extended in several other ways. First, one could extend the associational analysis we conduct, to see whether these differences matter when analyzing correlations with other development outcomes, such as those related to women’s welfare. Second, with the use of timestamps to map survey length and even the length of individual modules becoming more routine, it might be possible to account more formally for possible respondent fatigue using updated versions of these datasets.

### Implications

Measuring multiple domains of women’s empowerment accurately and consistently across data collection strategies is important to be able to track change over time and monitor progress toward national, regional, or global goals, such as the SDGs and CAADP. If we cannot collect comparable data on these indicators, it is difficult to draw meaningful conclusions about changes across time or differences across contexts. These findings prompt two areas for consideration: use of existing data and developing best practices for future measurement.

First, how can we use existing data? We cannot retroactively collect women’s empowerment data, and 2030 is quickly approaching. Therefore, we must be practical about using existing data. Based on our findings, we recommend that, despite the need for more frequent monitoring and coverage across countries, comparisons only be made within the same survey programs—which limits comparisons to surveys with the same question format and approach to survey design—and suggest caution when comparing outcomes from questions that are framed differently or from surveys that are designed differently. When selecting from available surveys, it may be most practical to select those with the longest time coverage in a given country to prioritize being able to observe time trends with the same approach.

Second, it is evident that more work needs to be done on building evidence and reaching consensus on best practices for measuring women’s empowerment. Disagreements over the “best” way to measure even very specific domains of women’s empowerment are not new (Acosta et al., 2020; Bernard et al., 2020; Seymour & Peterman, 2018). Our paper contributes to this ongoing debate by clarifying the consequences of aiming to piece together conclusions about the progress of women’s empowerment across disparate data sources. While we cannot always conclude how specific differences in survey and questionnaire design affect responses based on current data, it is clear they matter. One approach to mitigate these differences is through the development of standardized, cross-nationally comparable women’s empowerment metrics. The Women’s Empowerment Metric for National Statistical Systems (WEMNS) is one such effort to develop an index and component indicators that aim to be comparable across countries, time, and genders (Seymour et al., 2024; Sinharoy et al., 2023; Yount et al., 2023). As a relatively brief and multidimensional measure of women’s empowerment, it holds promise but is in its first phase of being implemented at scale, and results from that work should be reviewed before issuing broad recommendations.

Third, careful qualitative work accompanying the quantitative data collection can help in contextualizing findings and highlighting country-specific nuance (Schmid, 2022). Researchers should not expect that the same areas of decisionmaking will necessarily emerge from the qualitative and quantitative work and should take advantage of the flexibility of the qualitative approach to follow leads as they arise.

It is our hope that future research into these topics will help refine the measurement of women’s empowerment, a necessary first step to the more important but considerably more difficult task of catalyzing gender transformative change.

## Supplementary Information

Below is the link to the electronic supplementary material.

Supplementary file1 (DOCX 329 KB)

## Data Availability

Data is freely downloadable from the respective survey websites and do-files to replicate this analysis are available upon request.

## Footnotes

1 While a useful starting point, the SWPER index has been critiqued on its construction, and the need for a fuller psychometric assessment of the index before making comparisons pointed out (Yount et al., 2018).

2 Exceptions include Senegal, which did not implement a household roster, making it impossible to limit the sample by household type or relation to household head; Guatemala, where all documentation was in Spanish and where pilot data was known to be of poor quality; Liberia, where post-harmonization sample sizes were too small for inference; and Zambia, where the DHS did not have district-level identifiers, so we could not harmonize by geography.

3 Dual-headed households are those with at least one adult woman and one adult man.

4 We opted to group “input into few decisions” with “no input or control” to account for the fact that participation in only a few decisions over the past 12 months might not be sufficient for respondents to regard themselves as decision makers as defined by DHS. It follows that the results from the FTF surveys should, if anything, underestimate control over income relative to the DHS.

5 The FTF questionnaire is structured such that a respondent who says they (singular) did not participate in an activity is not asked the follow-up questions about inputs into decisionmaking and control over use of income arising from that activity. Since this is hard coded in the questionnaire, we were not able to relax this assumption for our analysis. It is possible that a respondent could still make decisions about an activity or control the income arising from it even if they themselves do not participate in the activity. In such cases, the FTF survey would tend to *underestimate* input into decisionmaking and control over use of income.

6 Household ownership of 7 consumer durables (a TV, refrigerator, phone, bicycle, car, cheap utensil and expensive utensil), number of sleeping rooms, quality of floor material and quality of toilet facility, and access to clean water and electricity are combined using Principal Component Analysis and the first component used to derive the asset weights. These were then used to construct a wealth indicator for each household. Based on the distribution of this wealth indicator in our sample we formed 7 categories: 10th, 20th, 30th, 40th, 50th and 60th percentiles and the 70th percentile and above.
