# Supplementary material for: Do Estimates of Women’s Control over Income and Decisionmaking Vary Across Nationally Representative Survey Programs?
Source: Soc Indic Res. 2025 Apr 25;179(1):95–122. doi: 10.1007/s11205-025-03605-x (PMC12321913; doi:10.1007/s11205-025-03605-x)
Supplement: Supplementary file 1 — Supplementary file1 (DOCX 329 KB) [file 11205_2025_3605_MOESM1_ESM.docx]

**Journal name:** Social Indicators Research

**Article title:** Why do estimates of women’s control over income and decisionmaking vary? An examination of nationally representative survey programs

# Appendix [online only]

## Details of the LSMS-ISA sampling strategy, respondent selection and placement of modules

LSMS-ISA rounds were selected based on proximity to the most recent FTF survey year in the three countries where the LSMS was available, Malawi, Tanzania and Uganda. As with the DHS, LSMS-ISA surveys are both nationally representative and issue multi-stage stratified random samples where enumeration areas are randomly selected with a probability proportional to their population. Households are then randomly selected within each enumeration area. The LSMS-ISA surveys often introduce a panel component and are considerably more complicated in design than the DHS; we abstract from much of this complexity by using single cross-sections in each country. We limit the LSMS-ISA sample to areas covered by the FTF survey in each country.

LSMS-ISA surveys usually require all household members to answer the household, education, and labor force modules, such that each person answers for themselves directly. However, for modules on agriculture, livestock or non-agricultural enterprises where decision-making questions are embedded, the respondent is usually either the household head or the person most familiar with the production activities in question. Depending on placement within the survey modules, questions on control over income in the LSMS surveys in Malawi, Tanzania and Uganda could be posed to the household head or most knowledgeable member, who is asked to list up to two household member IDs (Table 2). Questions on salary income of each adult household member in the Malawi survey could be responded to by any other household member. Unfortunately, since the control over income questions in LSMS-ISA are obtained from multiple modules, for whom the identity of respondent is not reported, it is not straightforward to estimate how often women are reporting for themselves.

For LSMS-ISA, a woman was classified as having "some/all input or control" if hers was one of the household member IDs mentioned by the respondent as having input into the use of income for any of multiple sources; otherwise, she was classified as having "no input or control". For the category of “other income” in Malawi and Uganda, a filtering question was applied, asking the respondent if their household had received income from any one of a set of other sources of income; if the respondent said no to any individual source, they were classified as not having control over that source of income.

In LSMS-ISA, questions on control over income were typically spread across the household and individual questionnaires (non-agricultural income sources; these modules were typically administered first) and the agriculture and livestock and fishery questionnaires (for income from crop sales, livestock and livestock products; these modules were typically administered either at the end of Day 1 or on Day 2 with the household).

## Appendix figures


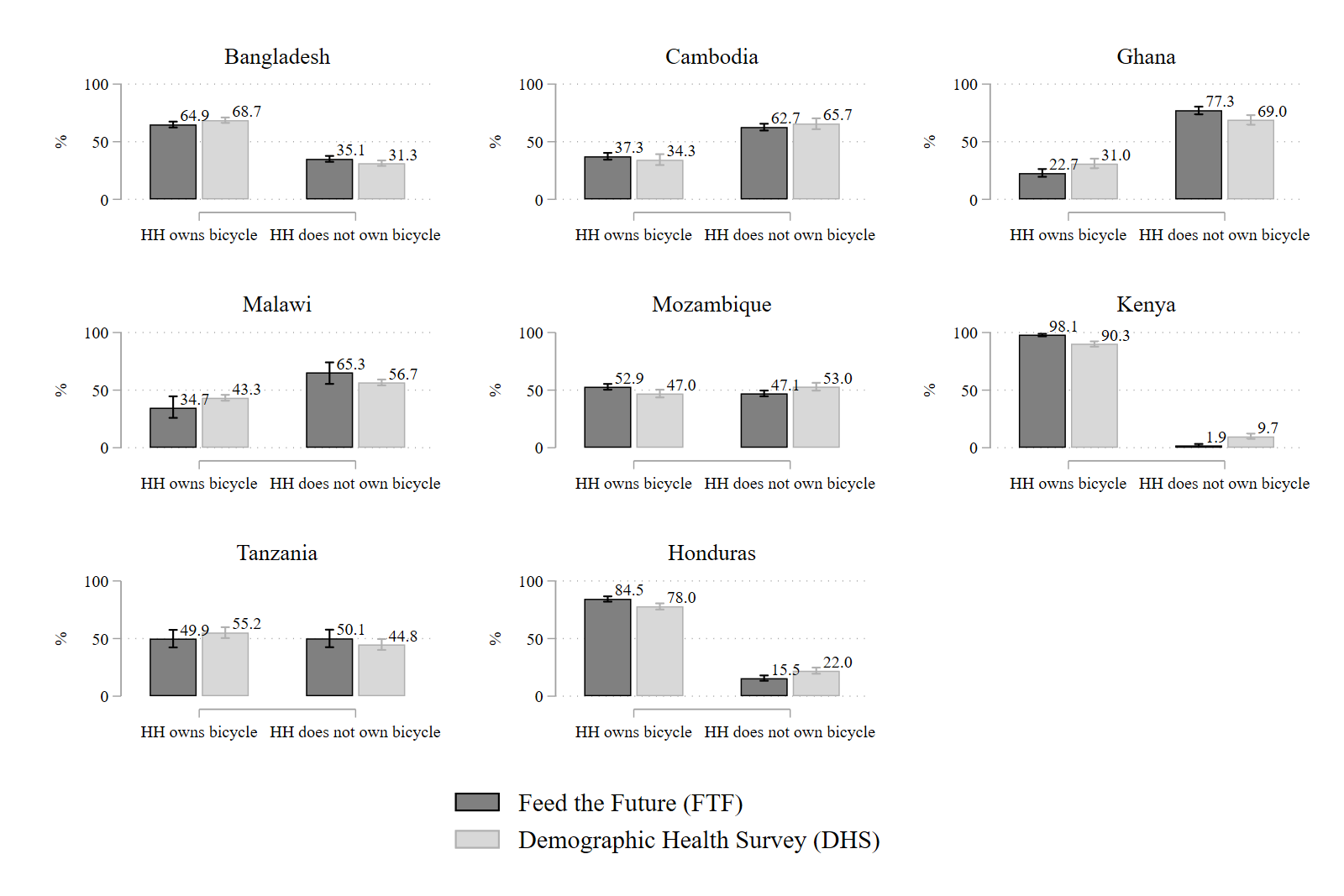


Figure A.1: Comparison of bicycle ownership in the DHS and FTF


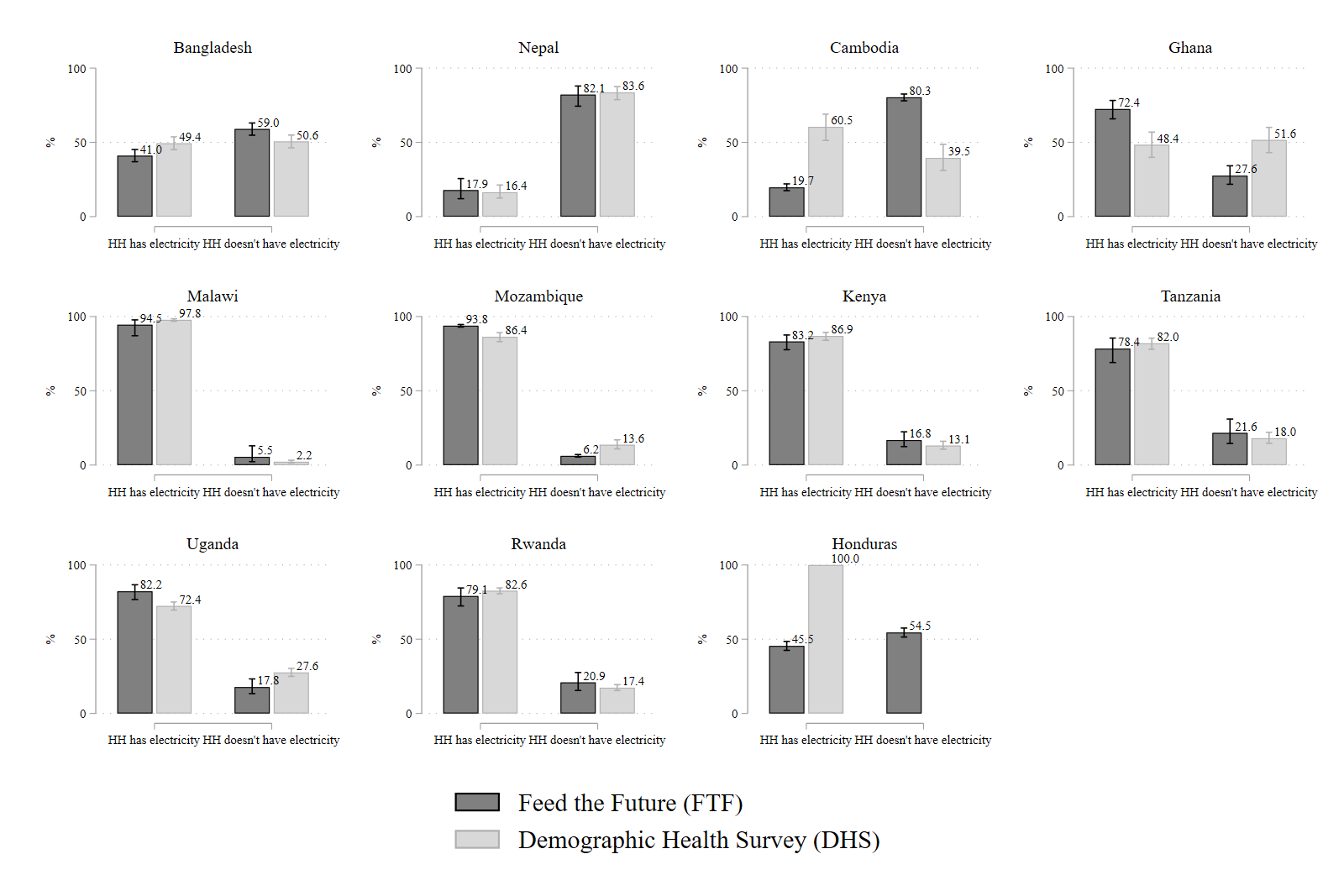


Figure A.2: Comparison of household access to electricity in the DHS and FTF

## Appendix tables

Table A.1: LSMS-ISA survey characteristics by country

| **Country** | **LSMS-ISA** | | |
| --- | --- | --- | --- |
|  | **Survey Month and Year** | **Sample frame** | **Post-harmonization sample size** |
| **Malawi** | December 2016-September 2017 | 4 regions | 414 |
| **Tanzania** | October 2014-September 2015 | 26 regions (including Dar es Salaam city) | 699 |
| **Uganda** | March 2015-March 2016 | Kampala District, Central excluding Kampala, Eastern, Western and Northern | 859 |
| Note: LSMS-ISA: Living Standards Measurement Surveys - Integrated Surveys on Agriculture. Authors' additions based on review of the questionnaires across the three survey programs. The achieved sample size refers to the final sample after harmonization across surveys (see text for details). Where information was unclear or inconsistent, we contacted the survey programs directly for more details. | | | |

Table A.2: Information on the set of languages used in each survey

| **Country** | **FTF** | | **DHS** | | **LSMS** | |
| --- | --- | --- | --- | --- | --- | --- |
|  | **Language** | **Placement of control over income and decisionmaking questions** | **Language** | **Placement of control over income and decisionmaking questions** | **Language** | **Placement of control over income questions** |
| **Bangladesh** | Bangla | End of a very lengthy questionnaire. For men, comes after lengthy modules on agriculture and livestock practices, non-food expenditures. For women, comes after lengthy modules on food consumption, health and illness, HH food consumption and food security, nutrition practices and services. | Bangla | Section 9 of 11, page 64 of 75. Comes after lengthy modules detailing reproductive history, knowledge and use of contraception, pregnancy and post-natal care, child immunization, heaalth and nutrition, marriage and sexual activity. |  |  |
| **Cambodia** | Khmer, Chinese, Chaam, Vietnamese,Thai and Lao. | Third from last module. Comes after roster, household consumption expenditure modules but before anthropometry and dietary diversity and module on child anthro and IYCF. Pages 41-55 of 68 page questionnaire (not including annexes). | Khmer | Section 9 of 11, page 64 of 75. Comes after lengthy modules detailing reproductive history, knowledge and use of contraception, pregnancy and post-natal care, child immunization, health and nutrition, marriage and sexual activity. |  |  |
| **Ghana** | Questionnaire was in English. Translation was standardized at training and pretesting, but no information on incorporating this into programming. | HH survey was conducted in multiple visits. WEAI was administered at the end of visit 1, after roster, HH hunger scale, dietary diversity, child diet and woman and child anthropometry but before HH consumption expenditure (which was in visit 2). Pages 32-41 of 60. | Questionnaire translated into Akan, Ga, Ewe, Nzema, Dagbani. | Section 9 of 11, page 64 of 75. Comes after lengthy modules detailing reproductive history, knowledge and use of contraception, pregnancy and post-natal care, child immunization, health and nutrition, marriage and sexual activity. |  |  |
| **Haiti** | Haitian Creole; no other translated versions are available. | Survey was split into two firms - one did HH and women's questionnaires, one did agriculture and WEAI. In the second, the WEAI comes after HH roster and a lengthy ag module on practices, extension etc. But overall tool is only 25 pages long (WEAI is 17-25) | French, Haitian Creole. | Section 8 of 10, page 48 of 59. Comes after lengthy modules on reproductive history, contraception, pregnancy and post-natal care, child immunization, health and nutrition, marriage and sexual activity and fertility preferences. |  |  |
| **Honduras** | Spanish. | WEAI comes after roster, lengthy ag and ag extension module, SES module, market and credit participation module, HH consumption. Page 27-30 of 42) | Spanish. | Section 8 of 10, page 48 of 59. Comes after lengthy modules on reproductive history, contraception, pregnancy and post-natal care, child immunization, health and nutrition, marriage and sexual activity and fertility preferences. |  |  |
| **Kenya** | Three native languages spoken by 10 percent or more of the population in the Zone of Influence. In northern Kenya, the questionnaire was translated into Kiswahili, Somali, and Turkana. Translations were programmed into tablets. | 88 page questionnaire. WEAI comes after HH roster, consumption expenditure module, household hunger scale. Pages 51-65 of 88. | Questionnaire available in 15 local languages (in addition to English): Borana, Embu, Kalenjin, Kamba, Kikuyu, Kisii, Luhya, Maragoli, Luo, Maasai, Meru, Mijikenda, Pokot, Somali, Swahili, and Turkana. | Section 9 of 11, page 64 of 75. Comes after lengthy modules detailing reproductive history, knowledge and use of contraception, pregnancy and post-natal care, child immunization, heaalth and nutrition, marriage and sexual activity. |  |  |
| **Malawi** | Translated into Chichewa and field tested. Translations were not programmed. | 24 page questionnaire - WEAI comes after HH roster, dwelling characteristics and HH hunger scale, all relatively short modules. Pages 8-19 of 24. | Questionnaire was available in English, Chichewa and Tumbuka. | Section 9 of 11, page 64 of 75. Comes after lengthy modules detailing reproductive history, knowledge and use of contraception, pregnancy and post-natal care, child immunization, heaalth and nutrition, marriage and sexual activity. | Questionnaire was in English. Key terms like ‘household’, ‘head of household’, ’occupation’ etc. were discussed during training and a table with translations of these terms was provided in Chichewa, Chitumbuka, and Chiyao. | Questions on income from crop sales and livestock products administered as part of the agriculture questionnaire - for crops, spread from around the middle of the tool (pages 36-38 of 98) to the end, for livestock, at the end (page 89 of 98). Questions on rental income, sale of assets etc towards the end of the household questionnaire (page 56 of 69). Panel households were to be administered the HH questionnaire in full on the first visit, followed by the agriculture questionnaire. Questions on control over salary income embedded in time use and labor modules in first quarter of household questionnaire. |
| **Mozambique** | The survey questionnaire was translated into Portuguese and loaded onto tablet computers. | <No information available> | Portuguese. | Section 8 of 10, page 48 of 59. Comes after lengthy modules on reproductive history, contraception, pregnancy and post-natal care, child immunization, health and nutrition, marriage and sexual activity and fertility preferences. |  |  |
| **Nepal** | *<No information provided>* | Short questionnaire - ~26 pages, WEAI is main component. Comes after HH roster and two short modules on dwelling characteristics and household hunger scale. Pages 9-21 of 26. | Nepali, Maithili, Bhojpuri. | Section 9 of 11, page 64 of 75. Comes after lengthy modules detailing reproductive history, knowledge and use of contraception, pregnancy and post-natal care, child immunization, heaalth and nutrition, marriage and sexual activity. |  |  |
| **Rwanda** | Translated into Kinyarwanda and incorporated into the data entry program. | 40 page questionnaire. WEAI appears after roster and demographics, dwelling characteristics and hh hunger scale. Pages 9-23 of 40. | Questionnaire available in Kinyarwanda and English. | Section 9 of 11, page 64 of 75. Comes after lengthy modules detailing reproductive history, knowledge and use of contraception, pregnancy and post-natal care, child immunization, heaalth and nutrition, marriage and sexual activity. |  |  |
| **Tanzania** | *<No information provided>* | Very short 14 page questionnaire. WEAI appears after roster, HH hunger scale and dietary diversity modules, all short. Pages 6-14. | Questionnaire available in Kiswahili and English. | Section 9 of 11, page 64 of 75. Comes after lengthy modules detailing reproductive history, knowledge and use of contraception, pregnancy and post-natal care, child immunization, heaalth and nutrition, marriage and sexual activity. | Survey produced in English and Kiswahili; no further details available. | Decisions on remittances and assistance programs asked in Household and Individual Questionnaire, administered first. Control over income from crop sales asked in agricultural questionnaire (implemented late in interview or potentially on Day 2). Questions on livestock and livestock products in the Livestock & Fishery questionnaire, implemented after the household questionnaire for those households engaged in these activities (implemented late in interview or potentially on Day 2). |
| **Uganda** | Questionnaire translated into Luganda, Luo, Runyankole-Rukiga, Lusoga, Lugisu, and Runyoro (combined with Rutoro), translations incorporated into the data entry program used for data collection in the households. | 58 page questionnaire. WEAI appears after HH roster, dwelling characteristics, HH hunger scale. Pages 10-24 of 58. | Questionnaire translated into Luganda, Luo, Lugbara, Ateso, Ngakarimojong, Runyankole/Rukiga, Runyoro/Rutoro, Lusoga. | Section 9 of 11, page 64 of 75. Comes after lengthy modules detailing reproductive history, knowledge and use of contraception, pregnancy and post-natal care, child immunization, heaalth and nutrition, marriage and sexual activity. | Survey only produced in English; no further details available. | Questions on use of income from non-agricultural sources are spread across sections in the household questionnaire, from pages 22-35 of 45. Questions on control over revenue from meat/eggs/milk are towards the end of the agriculture questionnaire, pages 32-34 of 39. |
| Note: FTF: Feed the Future, DHS: Demographic Health Surveys, LSMS-ISA: Living Standards Measurement Surveys - Integrated Surveys on Agriculture. Authors' additions based on review of the questionnaires across the three survey programs. Where information was unclear or inconsistent, we contacted the survey programs directly for more details. | | | | | | |

Table A.3: Descriptive statistics for key individual and household characteristics across surveys, Bangladesh, Nepal, Cambodia

|  | Bangladesh | | Nepal | | Cambodia | |
| --- | --- | --- | --- | --- | --- | --- |
|  | FTF (N=4207) | DHS (N=7708) | FTF (N=592) | DHS (N=1663) | FTF (N=899) | DHS (N=1457) |
|  | Mean/prop. (SD) | Mean/prop. (SD) | Mean/prop. (SD) | Mean/prop. (SD) | Mean/prop. (SD) | Mean/prop. (SD) |
| Current age | 38.68 (11.65) | 33.29 (8.57) | 41.67 (12.95) | 36.09 (7.71) | 42.97 (13.05) | 35.56 (8.23) |
| Primary education or more | 0.41 (0.49) | 0.47 (0.5) | 0.21 (0.41) | 0.28 (0.45) | 0.69 (0.46) | 0.23 (0.42) |
| Number of household members (listed) | 4.42 (1.62) | 4.94 (1.9) | 5.11 (1.97) | 5.14 (2.17) | 4.74 (1.99) | 4.83 (1.73) |
| Household with improved roof | 0.98 (0.15) | 0.98 (0.15) | 0.86 (0.35) | 0.89 (0.32) | 0.96 (0.19) | 0.9 (0.29) |
| Household has low quality water supply | 0.15 (0.36) | 0.97 (0.16) | 0.39 (0.49) | 0.45 (0.5) | 0.71 (0.46) | 0.8 (0.4) |
| Household has medium quality water supply | 0.59 (0.49) | 0.01 (0.1) | 0.39 (0.49) | 0.27 (0.45) | 0.16 (0.36) | 0.09 (0.29) |
| Household has low quality toilet facility | 0.55 (0.5) | 0.26 (0.44) | 0.14 (0.35) | 0.07 (0.26) | 0.57 (0.5) | 0.54 (0.5) |
| Household has medium quality toilet facility | 0.43 (0.5) | 0.69 (0.46) | 0.35 (0.48) | 0.29 (0.45) | 0.34 (0.47) | 0.11 (0.31) |
| Household has low quality floor | 0.85 (0.36) | 0.83 (0.38) | 0.8 (0.4) | 0.75 (0.43) | 0.11 (0.31) | 0.06 (0.24) |
| Household has medium quality floor | 0.15 (0.36) | 0 (0.06) | 0.2 (0.4) | 0.00 (0.00) | 0.78 (0.41) | 0.79 (0.4) |
| Household owns a TV | 0.31 (0.46) | 0.32 (0.47) |  | 0.39 (0.49) | 0.58 (0.49) | 0.53 (0.5) |
| Household owns a fridge | 0.01 (0.11) | 0.1 (0.3) |  | 0.11 (0.31) | 0.58 (0.49) | 0.02 (0.15) |
| Household owns a phone | 0.88 (0.32) | 0.89 (0.32) |  | 0.95 (0.22) | 0.73 (0.45) | 0.86 (0.34) |
| Household owns a car | 0.01 (0.07) | 0.01 (0.08) |  | 0.03 (0.18) | 0.04 (0.21) | 0.11 (0.31) |
| Household owns a bicycle | 0.31 (0.46) | 0.33 (0.47) |  | 0.42 (0.49) | 0.63 (0.48) | 0.65 (0.48) |
| Household owns at least one cheap utensil | 0.98 (0.15) | 0.67 (0.47) |  | 0.97 (0.17) | 0.97 (0.18) | 0.96 (0.2) |
| Household owns at least one expensive utensil | 0.25 (0.43) | 0.1 (0.3) |  | 0.4 (0.49) | 0.66 (0.47) | 0.64 (0.48) |
| Household has access to electricity | 0.59 (0.49) | 0.5 (0.5) | 0.83 (0.38) | 0.82 (0.38) | 0.81 (0.4) | 0.36 (0.48) |
| Household uses improved cooking fuel | 0.05 (0.21) | 0.04 (0.19) | 0.16 (0.37) | 0.23 (0.42) | 0.07 (0.26) | 0.06 (0.24) |
| Household have less than 1 bedroom | 0.33 (0.47) | 0.3 (0.46) | 0.25 (0.43) | 0.22 (0.41) | 0.78 (0.41) | 0.72 (0.45) |
| Household has 2 bedrooms | 0.41 (0.49) | 0.4 (0.49) | 0.38 (0.49) | 0.41 (0.49) | 0.15 (0.36) | 0.21 (0.41) |
| Household in the poorest quantile | 0.02 (0.14) | 0.08 (0.28) |  | 0.00 (0.06) | 0.01 (0.12) | 0.02 (0.13) |

Source: Authors’ calculations.

Table A.4: Descriptive statistics for key individual and household characteristics across surveys, Ghana, Malawi, Mozambique

|  | Ghana | | Malawi | |  | Mozambique | |
| --- | --- | --- | --- | --- | --- | --- | --- |
|  | FTF (N=2860) | DHS (N=2060) | FTF (N=694) | DHS (N=3024) | LSMS (N=414) | FTF (N=1916) | DHS (N=2718) |
|  | Mean/prop. (SD) | Mean/prop. (SD) | Mean/prop. (SD) | Mean/prop. (SD) | Mean/prop. (SD) | Mean/prop. (SD) | Mean/prop. (SD) |
| Current age | 37.92 (13.33) | 33.45 (8.26) | 37.00 (14.92) | 31.12 (8.62) | 38.77 (16.27) | 40.69 (14.48) | 31.06 (8.76) |
| Primary education or more | 0.07 (0.26) | 0.28 (0.45) | 0.1 (0.3) | 0.18 (0.39) | 0.09 (0.29) | 0.12 (0.32) | 0.13 (0.33) |
| Number of household members (listed) | 6.35 (3.17) | 6.2 (3.16) | 5.18 (1.8) | 5.18 (1.91) | 5.29 (2.09) | 5.07 (1.99) | 5.26 (2.08) |
| Household with improved roof | 0.62 (0.49) | 0.85 (0.36) | 0.26 (0.44) | 0.3 (0.46) | 0.39 (0.49) | 0.19 (0.39) | 0.21 (0.41) |
| Household has low quality water supply | 0.73 (0.45) | 0.69 (0.46) | 0.89 (0.31) | 0.88 (0.33) | 0.92 (0.27) | 0.64 (0.48) | 0.69 (0.46) |
| Household has medium quality water supply | 0.18 (0.38) | 0.21 (0.41) | 0.1 (0.3) | 0.11 (0.31) | 0.06 (0.23) | 0.32 (0.47) | 0.26 (0.44) |
| Household has low quality toilet facility | 0.83 (0.37) | 0.58 (0.49) | 0.16 (0.37) | 0.14 (0.34) | 0.12 (0.33) | 0.93 (0.25) | 0.73 (0.44) |
| Household has medium quality toilet facility | 0.15 (0.36) | 0.39 (0.49) | 0.84 (0.37) | 0.86 (0.34) | 0.88 (0.33) | 0.03 (0.17) | 0.26 (0.44) |
| Household has low quality floor | 0.3 (0.46) | 0.13 (0.34) | 0.89 (0.32) | 0.89 (0.31) | 0.83 (0.38) | 0.88 (0.32) | 0.59 (0.49) |
| Household has medium quality floor | 0.68 (0.47) | 0 (0) | 0.11 (0.32) | 0 (0) | 0.17 (0.38) | 0.09 (0.29) | 0.27 (0.44) |
| Household owns a TV | 0.19 (0.39) | 0.4 (0.49) | 0.04 (0.2) | 0.05 (0.21) | 0.03 (0.18) | 0.08 (0.27) | 0.13 (0.34) |
| Household owns a fridge | 0.06 (0.24) | 0.17 (0.37) | 0.01 (0.08) | 0.01 (0.12) | 0.01 (0.08) | 0.02 (0.14) | 0.06 (0.24) |
| Household owns a phone | 0.6 (0.49) | 0.82 (0.39) | 0.47 (0.5) | 0.54 (0.5) | 0.45 (0.5) | 0.75 (0.43) | 0.27 (0.44) |
| Household owns a car | 0.02 (0.13) | 0.06 (0.23) | 0.01 (0.1) | 0.01 (0.11) | 0 (0.05) | 0 (0.05) | 0.02 (0.15) |
| Household owns a bicycle | 0.76 (0.43) | 0.69 (0.46) | 0.51 (0.5) | 0.57 (0.5) | 0.52 (0.5) | 0.47 (0.5) | 0.53 (0.5) |
| Household owns at least one cheap utensil | 0.94 (0.23) | 0.99 (0.1) | 0.75 (0.44) | 0.95 (0.22) | 0.78 (0.42) | 0.84 (0.37) | 0.81 (0.4) |
| Household owns at least one expensive utensil | 0.33 (0.47) | 0.48 (0.5) | 0.1 (0.3) | 0.05 (0.21) | 0.02 (0.15) | 0.09 (0.28) | 0.11 (0.31) |
| Household has access to electricity | 0.26 (0.44) | 0.5 (0.5) | 0.03 (0.18) | 0.02 (0.15) | 0.03 (0.17) | 0.09 (0.28) | 0.15 (0.35) |
| Household uses improved cooking fuel | 0.02 (0.15) | 0.06 (0.24) | 0.00 (0.05) | 0.00 (0.03) | 0.00 (0.00) | 0.00 (0.00) | 0 (0.05) |
| Household have less than 1 bedroom | 0.11 (0.32) | 0.31 (0.46) | 0.3 (0.46) | 0.4 (0.49) | 0.3 (0.46) | 0.15 (0.36) | 0.37 (0.48) |
| Household has 2 bedrooms | 0.23 (0.42) | 0.33 (0.47) | 0.45 (0.5) | 0.39 (0.49) | 0.37 (0.48) | 0.37 (0.48) | 0.41 (0.49) |
| Household in the poorest quantile | 0.02 (0.15) | 0.01 (0.1) | 0.13 (0.34) | 0.07 (0.25) | 0.13 (0.34) | 0.11 (0.31) | 0.28 (0.45) |

Source: Authors’ calculations.

Table A.5: Descriptive statistics for key individual and household characteristics across surveys, Kenya, Tanzania, Uganda

|  | Kenya | | Tanzania | | | Uganda | | |
| --- | --- | --- | --- | --- | --- | --- | --- | --- |
|  | FTF (N=1183) | DHS (N=2746) | FTF (N=507) | DHS (N=1659) | LSMS (N=699) | FTF (N=504) | DHS (N=3998) | LSMS (N=859) |
|  | Mean/prop. (SD) | Mean/prop. (SD) | Mean/prop. (SD) | Mean/prop. (SD) | Mean/prop. (SD) | Mean/prop. (SD) | Mean/prop. (SD) | Mean/prop. (SD) |
| Current age | 36.93 (14.15) | 31.39 (7.97) | 38.21 (11.76) | 33.64 (8.64) | 38.31 (12.78) | 34.31 (11.59) | 31.48 (8.56) | 40 (14.06) |
| Primary education or more | 0.2 (0.4) | 0.2 (0.4) | 0.67 (0.47) | 0.74 (0.44) | 0.63 (0.48) | 0.27 (0.45) | 0.36 (0.48) | 0.36 (0.48) |
| Number of household members (listed) | 5.4 (1.97) | 6.21 (2.48) | 5.65 (2.26) | 5.61 (2.43) | 5.58 (2.38) | 6.09 (2.45) | 5.64 (2.35) | 6.16 (2.78) |
| Household with improved roof | 0.46 (0.5) | 0 (0.05) | 0.86 (0.35) | 0.82 (0.39) | 0.84 (0.36) | 0.74 (0.44) | 0.69 (0.46) | 0.76 (0.43) |
| Household has low quality water supply | 0.56 (0.5) | 0.58 (0.49) | 0.35 (0.48) | 0.29 (0.46) | 0.34 (0.47) | 0.72 (0.45) | 0.66 (0.47) | 0.64 (0.48) |
| Household has medium quality water supply | 0.25 (0.44) | 0.23 (0.42) | 0.09 (0.28) | 0.49 (0.5) | 0.06 (0.25) | 0.24 (0.43) | 0.3 (0.46) | 0.29 (0.46) |
| Household has low quality toilet facility | 0.08 (0.27) | 0.57 (0.49) | 0.35 (0.48) | 0.38 (0.49) | 0.34 (0.48) | 0.1 (0.3) | 0.5 (0.5) | 0.53 (0.5) |
| Household has medium quality toilet facility | 0.37 (0.48) | 0.41 (0.49) | 0.33 (0.47) | 0.45 (0.5) | 0.32 (0.47) | 0.87 (0.34) | 0.49 (0.5) | 0.46 (0.5) |
| Household has low quality floor | 0.77 (0.42) | 0.73 (0.44) | 0.33 (0.47) | 0.44 (0.5) | 0.34 (0.47) | 0.73 (0.45) | 0.71 (0.45) | 0.68 (0.47) |
| Household has medium quality floor | 0.21 (0.41) | 0 (0.05) | 0.66 (0.47) | 0 (0) | 0.66 (0.47) | 0.24 (0.43) | 0.01 (0.09) | 0.31 (0.46) |
| Household owns a TV | 0.13 (0.34) | 0.16 (0.37) | 0.39 (0.49) | 0.27 (0.44) | 0.35 (0.48) |  | 0.12 (0.32) | 0.12 (0.32) |
| Household owns a fridge | 0.03 (0.16) | 0.03 (0.18) | 0.24 (0.43) | 0.15 (0.36) | 0.23 (0.42) |  | 0.03 (0.16) | 0.03 (0.16) |
| Household owns a phone | 0.58 (0.49) | 0.69 (0.46) | 0.87 (0.34) | 0.88 (0.32) | 0.86 (0.34) |  | 0.8 (0.4) | 0.81 (0.39) |
| Household owns a car | 0.01 (0.12) | 0.03 (0.16) | 0.04 (0.2) | 0.05 (0.22) | 0.04 (0.2) |  | 0.03 (0.17) | 0.02 (0.15) |
| Household owns a bicycle | 0.02 (0.15) | 0.1 (0.3) | 0.5 (0.5) | 0.51 (0.5) | 0.48 (0.5) |  | 0.42 (0.49) | 0.48 (0.5) |
| Household owns at least one cheap utensil | 0.71 (0.45) | 0.72 (0.45) | 1 (0) | 0.87 (0.33) | 1 (0) |  | 0.97 (0.16) | 0.98 (0.14) |
| Household owns at least one expensive utensil | 0.1 (0.3) | 0.12 (0.33) | 0.21 (0.41) | 0.22 (0.41) | 0.19 (0.39) |  | 0.31 (0.46) | 0.16 (0.37) |
| Household has access to electricity | 0.16 (0.36) | 0.17 (0.37) | 0.41 (0.49) | 0.29 (0.46) | 0.39 (0.49) | 0.19 (0.39) | 0.25 (0.43) | 0.1 (0.29) |
| Household uses improved cooking fuel | 0.01 (0.08) | 0.01 (0.12) | 0.04 (0.2) | 0.03 (0.16) | 0.04 (0.19) | 0 (0.04) | 0 (0.06) | 0 (0.03) |
| Household have less than 1 bedroom | 0.63 (0.48) | 0.62 (0.48) | 0.09 (0.29) | 0.27 (0.44) | 0.1 (0.3) | 0.33 (0.47) | 0.39 (0.49) | 0.23 (0.42) |
| Household has 2 bedrooms | 0.25 (0.43) | 0.28 (0.45) | 0.3 (0.46) | 0.41 (0.49) | 0.28 (0.45) | 0.36 (0.48) | 0.33 (0.47) | 0.3 (0.46) |
| Household in the poorest quantile | 0.12 (0.32) | 0.29 (0.45) | 0.04 (0.19) | 0.07 (0.25) | 0.03 (0.18) |  | 0.07 (0.26) | 0.06 (0.23) |

Source: Authors’ calculations.

Table A.6: Descriptive statistics for key individual and household characteristics across surveys, Rwanda, Haiti, Honduras

|  | Rwanda | | Haiti | | Honduras | |
| --- | --- | --- | --- | --- | --- | --- |
|  | FTF survey (N=764) | DHS Survey (N=5693) | FTF survey (N=413) | DHS Survey (N=2583) | FTF survey (N=1893) | DHS Survey (N=2615) |
|  | Mean/prop. (SD) | Mean/prop. (SD) | Mean/prop. (SD) | Mean/prop. (SD) | Mean/prop. (SD) | Mean/prop. (SD) |
| Current age | 38.59 (12.78) | 33.47 (7.69) | 41.74 (15.31) | 34.98 (8.21) | 40.91 (14.15) | 33.34 (8.4) |
| Primary education or more | 0.56 (0.5) | 0.35 (0.48) | 0.27 (0.45) | 0.43 (0.5) | 0.72 (0.45) | 0.35 (0.48) |
| Number of household members (listed) | 5.16 (1.84) | 5.1 (1.83) | 4.61 (2.1) | 5.39 (2.23) | 5.43 (2.22) | 5.49 (2.19) |
| Household with improved roof | 0.99 (0.08) | 1 (0.05) |  | 0.96 (0.2) | 0.99 (0.08) | 0.99 (0.08) |
| Household has low quality water supply | 0.37 (0.48) | 0.31 (0.46) |  | 0.31 (0.46) | 0.11 (0.31) | 0.19 (0.4) |
| Household has medium quality water supply | 0.56 (0.5) | 0.63 (0.48) |  | 0.34 (0.48) | 0.04 (0.19) | 0.71 (0.45) |
| Household has low quality toilet facility | 0.32 (0.47) | 0.25 (0.43) |  | 0.3 (0.46) | 0.23 (0.42) | 0.31 (0.46) |
| Household has medium quality toilet facility | 0.66 (0.47) | 0.74 (0.44) |  | 0.59 (0.49) | 0.33 (0.47) | 0.48 (0.5) |
| Household has low quality floor | 0.79 (0.41) | 0.81 (0.39) |  | 0.32 (0.46) | 0.45 (0.5) | 0.44 (0.5) |
| Household has medium quality floor | 0.21 (0.41) | 0 (0) |  | 0 (0.04) | 0.46 (0.5) | 0.43 (0.5) |
| Household owns a TV |  | 0.09 (0.28) |  | 0.41 (0.49) |  | 0.4 (0.49) |
| Household owns a fridge |  | 0.01 (0.09) |  | 0.13 (0.34) |  | 0.24 (0.43) |
| Household owns a phone |  | 0.64 (0.48) |  | 0.84 (0.36) |  | 0.78 (0.41) |
| Household owns a car |  | 0.01 (0.1) |  | 0.06 (0.24) |  | 0.12 (0.32) |
| Household owns a bicycle |  | 0.2 (0.4) |  | 0.11 (0.31) |  | 0.2 (0.4) |
| Household owns at least one cheap utensil |  | 0.7 (0.46) |  | 0.96 (0.19) |  | 0.98 (0.13) |
| Household owns at least one expensive utensil |  | 0.03 (0.17) |  | 0.18 (0.39) |  | 0.18 (0.39) |
| Household has access to electricity | 0.23 (0.42) | 0.19 (0.39) |  | 0.5 (0.5) | 0.54 (0.5) | 0 (0) |
| Household uses improved cooking fuel | 0 (0) | 0 (0.04) |  | 0.05 (0.22) | 0.02 (0.15) | 0.06 (0.23) |
| Household have less than 1 bedroom | 0.14 (0.35) | 0.23 (0.42) |  | 0.44 (0.5) | 0.46 (0.5) | 0.51 (0.5) |
| Household has 2 bedrooms | 0.43 (0.5) | 0.47 (0.5) |  | 0.38 (0.49) | 0.38 (0.49) | 0.36 (0.48) |
| Household in the poorest quantile |  | 0.11 (0.31) |  | 0.07 (0.25) |  | 0.08 (0.27) |

Source: Authors’ calculations.

Table A.7: Full text of questions, respondents and responses used to create indicators in LSMS-ISA

| **Measure: Control over income from any source** | |
| --- | --- |
| **Question/response options** | **Respondent** |
| **Malawi** A. Who in your household kept/ decided what to do with the earnings from [ACTIVITY]?; where [ACTIVITY] is crop sales, livestock products, rental income, sale of assets etc.  B. Who in the household controls/ decides on the use of your salary payment?  Response options: Up to two IDs from the HH roster. | A. The household head or most informed adult household member.  B. Asked of all persons aged 5 years and older that are not among the selected respondents for the individual-level questionnaire; anyone could respond on behalf of the individual. |
| **Tanzania**   Who in your household decided what to do with these [ACTIVITY] earnings?; where [ACTIVITY] includes crop sales, livestock and livestock product sales.  Response options: Up to two IDs from the HH roster. | The household head or most informed adult household member. |
| **Uganda** A. Who controls the revenue from this [PRODUCT]?; where [PRODUCT] includes meat, eggs, milk.  B. Who in the household decides on the use of earnings from this enterprise/from [SOURCE]; where [SOURCE] includes 11 sources of earned income excluding livestock product sales.  Response options: Up to two IDs from the HH roster. | The household head or most informed adult household member. |
| **Approach to harmonization:** Converted into two response categories: no input or control and some/all input or control. For LSMS-ISA: a woman was classified as having "some/all input or control" if theirs was one of the household member IDs mentioned by the respondent; otherwise, they were classified as having "no input or control". In both the FTF and LSMS-ISA, if the respondent indicated some/all input or control over any one component, she was classified as having some/all input or control overall. | |

Table A.8: Association of control over income with individual and household characteristics: Asia

|  | Bangladesh | | Nepal | | Cambodia | |
| --- | --- | --- | --- | --- | --- | --- |
|  | FTF | DHS | FTF | DHS | FTF | DHS |
|  | b/se | b/se | b/se | b/se | b/se | b/se |
|  | (1) | (2) | (3) | (4) | (5) | (6) |
| Current age | 0.003^***^ | 0.004^***^ | 0.000 | -0.003 | 0.000 | -0.003^***^ |
|  | (0.001) | (0.001) | (0.001) | (0.002) | (0.000) | (0.001) |
| Primary education or more | 0.010 | -0.010 | -0.001 | 0.043 | 0.013 | 0.011 |
|  | (0.013) | (0.019) | (0.018) | (0.031) | (0.010) | (0.011) |
| Household size | 0.009^***^ | -0.016^***^ | 0.012 | -0.022^***^ | -0.002 | 0.013^**^ |
|  | (0.003) | (0.004) | (0.008) | (0.006) | (0.002) | (0.006) |
| Wealth Index: 2nd quantile | 0.063 | 0.032 |  | -0.066 | 0.003 | 0.046^**^ |
|  | (0.042) | (0.022) |  | (0.068) | (0.018) | (0.018) |
| Wealth Index: 3rd quantile | 0.063 | 0.045^*^ |  | 0.036 | -0.003 | 0.008 |
|  | (0.040) | (0.024) |  | (0.057) | (0.020) | (0.019) |
| Wealth Index: 4th quantile | 0.090^**^ | 0.008 |  | 0.041 | 0.011 | 0.006 |
|  | (0.040) | (0.026) |  | (0.044) | (0.016) | (0.023) |
| Wealth Index: 5th quantile | 0.078^*^ | 0.005 |  | 0.072^*^ | -0.005 | 0.029^*^ |
|  | (0.041) | (0.030) |  | (0.038) | (0.019) | (0.017) |
| Wealth Index: 6th quantile | 0.023 | -0.008 |  | 0.048 | 0.012 | -0.025 |
|  | (0.045) | (0.035) |  | (0.039) | (0.016) | (0.026) |
| Wealth Index: 7th quantile | 0.034 | -0.070^*^ |  | -0.009 | -0.021 | 0.001 |
|  | (0.051) | (0.037) |  | (0.058) | (0.025) | (0.027) |
| Constant | 0.719^***^ | 0.016 | 1.119^***^ | 1.079^***^ | 1.019^***^ | 1.029^***^ |
|  | (0.110) | (0.072) | (0.096) | (0.200) | (0.033) | (0.052) |
| Rural dummy | No | No | Yes | Yes | No | No |
| Division FE | Yes | Yes | Yes | Yes | Yes | Yes |
| Month FE | Yes | Yes | Yes | Yes | Yes | Yes |
| Interviewer FE | Yes | Yes | Yes | Yes | Yes | Yes |
| R-squared | 0.121 | 0.122 | 0.164 | 0.231 | 0.212 | 0.074 |
| Mean dependent variable | 0.87 | 0.33 | 0.97 | 0.64 | 0.98 | 0.96 |
| N | 4196 | 7294 | 592 | 1641 | 849 | 1353 |
| Notes: The dependent variable for these OLS regressions is a binary variable that is 0 if the individual has no input into or control over use of income from any source, and 1 if they have some or all input/control. Information on asset ownership was not available in the Nepal FTF, which is why household wealth quantiles are missing. | | | | | | |

Table A.9: Association of control over income with individual and household characteristics: West and South Africa

|  | Ghana | | Malawi | | | Mozambique | |
| --- | --- | --- | --- | --- | --- | --- | --- |
|  | FTF | DHS | FTF | LSMS | DHS | FTF | DHS |
|  | b/se | b/se | b/se | b/se | b/se | b/se | b/se |
|  | (1) | (2) | (3) | (4) | (5) | (6) | (7) |
| Current age | 0.001 | 0.007^***^ | 0.001^*^ | 0.001 | 0.000 | 0.001^*^ | 0.002 |
|  | (0.001) | (0.002) | (0.000) | (0.001) | (0.001) | (0.001) | (0.001) |
| Primary education or more | -0.033 | 0.100^***^ | 0.034 | 0.055 | 0.097^***^ | 0.060^*^ | 0.085^**^ |
|  | (0.048) | (0.029) | (0.023) | (0.050) | (0.026) | (0.036) | (0.040) |
| Household size | 0.005^**^ | -0.007^*^ | 0.008 | 0.014 | 0.007 | 0.005 | 0.002 |
|  | (0.003) | (0.004) | (0.006) | (0.014) | (0.005) | (0.006) | (0.005) |
| Wealth Index: 2nd quantile | -0.038 | -0.163^**^ | -0.030 | -0.087 | 0.081^***^ | 0.026 | 0.030 |
|  | (0.044) | (0.075) | (0.023) | (0.070) | (0.031) | (0.039) | (0.024) |
| Wealth Index: 3rd quantile | -0.024 | -0.039 | -0.008 | -0.049 | 0.077^**^ | -0.016 | 0.059^*^ |
|  | (0.036) | (0.058) | (0.020) | (0.058) | (0.035) | (0.040) | (0.031) |
| Wealth Index: 4th quantile | -0.047 | -0.056 | 0.001 | -0.058 | 0.106^**^ | -0.005 | 0.058 |
|  | (0.040) | (0.058) | (0.027) | (0.072) | (0.050) | (0.056) | (0.038) |
| Wealth Index: 5th quantile | -0.017 | -0.039 | 0.014 | -0.048 | 0.067 | -0.021 | 0.079 |
|  | (0.047) | (0.060) | (0.024) | (0.088) | (0.050) | (0.072) | (0.069) |
| Wealth Index: 6th quantile | 0.027 | -0.054 | -0.002 | 0.058 | 0.011 | -0.160 | 0.078^*^ |
|  | (0.049) | (0.052) | (0.028) | (0.111) | (0.111) | (0.107) | (0.046) |
| Wealth Index: 7th quantile | -0.022 | -0.125^***^ | -0.261 | 0.000 | -0.042 | -0.100 | 0.015 |
|  | (0.055) | (0.042) | (0.159) | (.) | (0.096) | (0.140) | (0.073) |
| Language mismatch |  |  | 0.037^*^ | -0.072 | -0.063 |  |  |
|  |  |  | (0.019) | (0.054) | (0.047) |  |  |
| Constant | 0.857^***^ | 0.392 | 0.879^***^ | 0.888^***^ | 0.678^***^ | 0.927^***^ | 0.079 |
|  | (0.130) | (0.318) | (0.056) | (0.135) | (0.108) | (0.062) | (0.106) |
| Rural dummy | Yes | Yes | No | No | No | Yes | Yes |
| Division FE | Yes | Yes | Yes | Yes | Yes | Yes | Yes |
| Month FE | Yes | Yes | Yes | Yes | Yes | Yes | Yes |
| Interviewer FE | Yes | Yes | No | Yes | Yes | No | Yes |
| R-squared | 0.196 | 0.249 | 0.033 | 0.383 | 0.221 | 0.079 | 0.287 |
| Mean dependent variable | 0.72 | 0.69 | 0.96 | 0.86 | 0.57 | 0.74 | 0.41 |
| N | 2860 | 2022 | 539 | 275 | 2913 | 1914 | 2673 |
| Notes: The dependent variable for these OLS regressions is a binary variable that is 0 if the individual has no input into or control over use of income from any source, and 1 if they have some or all input/control. | | | | | | | |

Table A.10: Association of control over income with individual and household characteristics: East Africa

|  | Kenya | | Tanzania | | | Uganda | | | Rwanda | |
| --- | --- | --- | --- | --- | --- | --- | --- | --- | --- | --- |
|  | FTF | DHS | FTF | LSMS | DHS | FTF | LSMS | DHS | FTF | DHS |
|  | b/se | b/se | b/se | b/se | b/se | b/se | b/se | b/se | b/se | b/se |
|  | (1) | (2) | (3) | (4) | (5) | (6) | (7) | (8) | (9) | (10) |
| Current age | 0.002^*^ | 0.002 | 0.001 | 0.001 | 0.001 | -0.000 | 0.002 | 0.005^***^ | -0.000 | -0.000 |
|  | (0.001) | (0.002) | (0.002) | (0.002) | (0.001) | (0.001) | (0.001) | (0.001) | (0.001) | (0.001) |
| Primary education or more | 0.074 | 0.112^***^ | 0.068^*^ | -0.098 | 0.068^**^ | -0.015 | 0.013 | 0.072^***^ | 0.011 | 0.030^**^ |
|  | (0.054) | (0.041) | (0.039) | (0.065) | (0.034) | (0.033) | (0.028) | (0.017) | (0.012) | (0.012) |
| Household size | 0.025^***^ | -0.005 | 0.011 | -0.010 | 0.003 | 0.001 | 0.006 | -0.009^*^ | 0.006^*^ | 0.001 |
|  | (0.009) | (0.007) | (0.008) | (0.010) | (0.005) | (0.005) | (0.004) | (0.005) | (0.003) | (0.004) |
| Wealth Index: 2nd quantile | 0.138^**^ | 0.032 | -0.103 | 0.056 | 0.003 |  | 0.063 | -0.045^*^ |  | 0.009 |
|  | (0.054) | (0.042) | (0.068) | (0.065) | (0.043) |  | (0.048) | (0.027) |  | (0.018) |
| Wealth Index: 3rd quantile | 0.057 | 0.167^***^ | -0.109 | 0.037 | 0.015 |  | 0.093^**^ | -0.037 |  | 0.022 |
|  | (0.063) | (0.061) | (0.071) | (0.088) | (0.042) |  | (0.046) | (0.026) |  | (0.018) |
| Wealth Index: 4th quantile | 0.072 | 0.114^**^ | -0.189^***^ | -0.055 | 0.054 |  | 0.063 | -0.044 |  | 0.020 |
|  | (0.069) | (0.052) | (0.067) | (0.128) | (0.038) |  | (0.061) | (0.028) |  | (0.024) |
| Wealth Index: 5th quantile | 0.155 | 0.077 | -0.184^**^ | 0.046 | 0.023 |  | 0.114^*^ | -0.014 |  | 0.021 |
|  | (0.101) | (0.077) | (0.083) | (0.106) | (0.044) |  | (0.065) | (0.033) |  | (0.024) |
| Wealth Index: 6th quantile | 0.121 | 0.067 | -0.110^*^ | 0.056 | 0.007 |  | 0.092 | -0.011 |  | 0.054^**^ |
|  | (0.103) | (0.071) | (0.064) | (0.142) | (0.041) |  | (0.075) | (0.034) |  | (0.026) |
| Wealth Index: 7th quantile | -0.055 | 0.021 | -0.337^**^ | -0.189 | 0.025 |  | 0.074 | 0.014 |  | 0.058^*^ |
|  | (0.098) | (0.080) | (0.146) | (0.195) | (0.040) |  | (0.070) | (0.038) |  | (0.030) |
| Language mismatch |  |  |  |  |  | -0.020 |  | -0.044^*^ |  |  |
|  |  |  |  |  |  | (0.020) |  | (0.025) |  |  |
| Constant | 0.421^***^ | 0.644^***^ | 1.172^***^ | 1.466^***^ | 0.803^***^ | 0.115^***^ | 0.900^***^ | 0.938^***^ | 0.923^***^ | 1.163^***^ |
|  | (0.094) | (0.133) | (0.408) | (0.331) | (0.171) | (0.024) | (0.054) | (0.153) | (0.049) | (0.051) |
| Rural dummy | Yes | Yes | Yes | Yes | Yes | Yes | Yes | Yes | Yes | Yes |
| Division FE | Yes | Yes | Yes | Yes | Yes | Yes | Yes | Yes | Yes | Yes |
| Month FE | Yes | Yes | Yes | Yes | Yes | Yes | Yes | Yes | Yes | Yes |
| Interviewer FE | No | Yes | Yes | Yes | Yes | No | No | Yes | No | Yes |
| R-squared | 0.115 | 0.273 | 0.235 | 0.384 | 0.114 | 0.140 | 0.132 | 0.234 | 0.048 | 0.092 |
| Mean dependent variable | 0.58 | 0.64 | 0.85 | 0.78 | 0.82 | 0.92 | 0.91 | 0.75 | 0.98 | 0.84 |
| N | 1183 | 1202 | 505 | 241 | 1613 | 498 | 786 | 3891 | 764 | 5464 |

Notes: The dependent variable for these OLS regressions is a binary variable that is 0 if the individual has no input into or control over use of income from any source, and 1 if they have some or all input/control. Information on asset ownership was not available in the Uganda FTF, which is why household wealth quantiles are missing.

Table A.11: Association of control over income with individual and household characteristics: Central America

|  | Haiti | | Honduras | |
| --- | --- | --- | --- | --- |
|  | FTF | DHS | FTF | DHS |
|  | b/se | b/se | b/se | b/se |
|  | (1) | (2) | (3) | (4) |
| Current age | -0.002 | 0.003^***^ | 0.001 | -0.000 |
|  | (0.001) | (0.001) | (0.001) | (0.001) |
| Primary education or more | -0.057 | -0.001 | 0.058 | 0.085^***^ |
|  | (0.042) | (0.016) | (0.037) | (0.020) |
| Household size | 0.018^***^ | -0.003 | 0.010 | -0.002 |
|  | (0.007) | (0.004) | (0.007) | (0.005) |
| Wealth Index: 2nd quantile |  | -0.021 | -0.130^*^ | -0.065^**^ |
|  |  | (0.025) | (0.074) | (0.027) |
| Wealth Index: 3rd quantile |  | -0.015 | -0.172^***^ | -0.040 |
|  |  | (0.030) | (0.055) | (0.029) |
| Wealth Index: 4th quantile |  | 0.032 | -0.083^*^ | -0.028 |
|  |  | (0.023) | (0.049) | (0.026) |
| Wealth Index: 5th quantile |  | 0.008 | -0.021 | -0.056^*^ |
|  |  | (0.023) | (0.050) | (0.034) |
| Wealth Index: 6th quantile |  | -0.002 | -0.056 | -0.059 |
|  |  | (0.023) | (0.051) | (0.037) |
| Wealth Index: 7th quantile |  | -0.034 | 0.001 | -0.054 |
|  |  | (0.026) | (0.053) | (0.037) |
| Interviewer is female |  |  | 0.087^**^ |  |
|  |  |  | (0.034) |  |
| Constant | 0.595^***^ | 0.892^***^ | 0.314^***^ | 0.947^***^ |
|  | (0.213) | (0.045) | (0.116) | (0.305) |
| Rural dummy | No | No | No | No |
| Division FE | Yes | Yes | Yes | Yes |
| Month FE | Yes | Yes | Yes | Yes |
| Interviewer FE | Yes | Yes | No | Yes |
| R-squared | 0.274 | 0.059 | 0.090 | 0.087 |
| Mean dependent variable | 0.91 | 0.89 | 0.55 | 0.80 |
| N | 402 | 2457 | 1885 | 2568 |
| Notes: The dependent variable for these OLS regressions is a binary variable that is 0 if the individual has no input into or control over use of income from any source, and 1 if they have some or all input/control. Information on asset ownership was not available in the Haiti FTF, which is why household wealth quantiles are missing. | | | | |

Table A.12: Association of decisionmaking with individual and household characteristics: Asia

|  | Bangladesh | | Nepal | | Cambodia | |
| --- | --- | --- | --- | --- | --- | --- |
|  | FTF | DHS | FTF | DHS | FTF | DHS |
|  | b/se | b/se | b/se | b/se | b/se | b/se |
|  | (1) | (2) | (3) | (4) | (5) | (6) |
| Current age | -0.000 | 0.002^***^ | -0.001 | -0.000 | 0.002^**^ | -0.009^***^ |
|  | (0.001) | (0.001) | (0.002) | (0.002) | (0.001) | (0.001) |
| Primary education or more | 0.025^*^ | 0.038^***^ | 0.012 | 0.072^**^ | 0.010 | -0.018 |
|  | (0.014) | (0.013) | (0.033) | (0.031) | (0.025) | (0.023) |
| Household size | -0.009^**^ | -0.018^***^ | -0.023^***^ | -0.027^***^ | -0.022^***^ | 0.032^***^ |
|  | (0.004) | (0.004) | (0.006) | (0.006) | (0.006) | (0.007) |
| Wealth Index: 2nd quantile | -0.034 | 0.001 |  | -0.138 | 0.136^**^ | -0.063 |
|  | (0.042) | (0.020) |  | (0.083) | (0.057) | (0.045) |
| Wealth Index: 3rd quantile | 0.008 | 0.012 |  | 0.017 | 0.044 | -0.099^**^ |
|  | (0.043) | (0.025) |  | (0.061) | (0.053) | (0.050) |
| Wealth Index: 4th quantile | 0.083^*^ | 0.003 |  | 0.011 | 0.075 | -0.065 |
|  | (0.043) | (0.023) |  | (0.049) | (0.049) | (0.043) |
| Wealth Index: 5th quantile | 0.087^**^ | 0.016 |  | 0.034 | 0.041 | -0.038 |
|  | (0.043) | (0.022) |  | (0.052) | (0.048) | (0.032) |
| Wealth Index: 6th quantile | 0.103^**^ | -0.024 |  | 0.020 | 0.093^**^ | -0.108^**^ |
|  | (0.050) | (0.037) |  | (0.053) | (0.044) | (0.048) |
| Wealth Index: 7th quantile | 0.066 | 0.044 |  | -0.064 | 0.043 | 0.004 |
|  | (0.062) | (0.034) |  | (0.051) | (0.049) | (0.031) |
| Constant | 0.814^***^ | 1.016^***^ | 0.655^***^ | 0.421^**^ | 0.964^***^ | 1.254^***^ |
|  | (0.180) | (0.064) | (0.135) | (0.194) | (0.094) | (0.159) |
| Rural dummy | No | No | Yes | Yes | No | No |
| Division FE | Yes | Yes | Yes | Yes | Yes | Yes |
| Month FE | Yes | Yes | Yes | Yes | Yes | Yes |
| Interviewer FE | Yes | Yes | Yes | Yes | Yes | Yes |
| R-squared | 0.421 | 0.169 | 0.335 | 0.152 | 0.563 | 0.116 |
| Mean dependent variable | 0.60 | 0.64 | 0.79 | 0.56 | 0.68 | 0.84 |
| N | 4196 | 7708 | 586 | 1663 | 899 | 1457 |
| Notes: The dependent variable for these OLS regressions is a binary variable that is 0 if the individual does not take any decisions, and 1 if they take decisions either solely or jointly. Information on asset ownership was not available in the Nepal FTF, which is why household wealth quantiles are missing. | | | | | | |

Table A.13: Association of decisionmaking with individual and household characteristics: West and South Africa

|  | Ghana | | Malawi | | Mozambique | |
| --- | --- | --- | --- | --- | --- | --- |
|  | FTF | DHS | FTF | DHS | FTF | DHS |
|  | b/se | b/se | b/se | b/se | b/se | b/se |
|  | (1) | (2) | (3) | (4) | (5) | (6) |
| Current age | -0.002^***^ | 0.008^***^ | 0.005^***^ | 0.001 | 0.002^**^ | 0.001 |
|  | (0.001) | (0.001) | (0.001) | (0.002) | (0.001) | (0.001) |
| Primary education or more | -0.030 | 0.060^*^ | 0.052 | 0.046^**^ | 0.067 | 0.106^***^ |
|  | (0.044) | (0.033) | (0.095) | (0.021) | (0.047) | (0.038) |
| Household size | 0.004 | -0.014^***^ | 0.010 | 0.010 | 0.009 | -0.000 |
|  | (0.003) | (0.004) | (0.009) | (0.006) | (0.007) | (0.005) |
| Wealth Index: 2nd quantile | -0.082 | -0.027 | 0.058 | 0.049 | 0.004 | 0.035 |
|  | (0.054) | (0.070) | (0.069) | (0.033) | (0.043) | (0.025) |
| Wealth Index: 3rd quantile | -0.043 | 0.016 | 0.074 | 0.067^*^ | -0.040 | 0.037 |
|  | (0.049) | (0.052) | (0.058) | (0.036) | (0.047) | (0.033) |
| Wealth Index: 4th quantile | 0.025 | 0.057 | 0.171^*^ | 0.060 | -0.035 | 0.073^*^ |
|  | (0.052) | (0.046) | (0.096) | (0.045) | (0.073) | (0.039) |
| Wealth Index: 5th quantile | 0.052 | 0.089 | 0.106 | 0.102^*^ | 0.003 | 0.093^*^ |
|  | (0.062) | (0.055) | (0.113) | (0.060) | (0.092) | (0.049) |
| Wealth Index: 6th quantile | 0.097 | -0.020 | -0.048 | -0.056 | -0.090 | -0.007 |
|  | (0.063) | (0.052) | (0.227) | (0.083) | (0.120) | (0.051) |
| Wealth Index: 7th quantile | 0.060 | -0.051 | 0.498^***^ | -0.129 | 0.064 | -0.061 |
|  | (0.062) | (0.047) | (0.124) | (0.098) | (0.160) | (0.065) |
| Language mismatch |  |  | -0.400^***^ | -0.105^**^ |  |  |
|  |  |  | (0.057) | (0.048) |  |  |
| Constant | 0.562^***^ | 0.832^***^ | 0.086 | 0.514^***^ | 0.433^***^ | 0.212^*^ |
|  | (0.150) | (0.111) | (0.107) | (0.077) | (0.076) | (0.128) |
| Rural dummy | Yes | Yes | No | No | Yes | Yes |
| Division FE | Yes | Yes | Yes | Yes | Yes | Yes |
| Month FE | Yes | Yes | Yes | Yes | Yes | Yes |
| Interviewer FE | Yes | Yes | No | Yes | No | Yes |
| R-squared | 0.228 | 0.271 | 0.073 | 0.237 | 0.023 | 0.288 |
| Mean dependent variable | 0.39 | 0.66 | 0.42 | 0.50 | 0.51 | 0.48 |
| N | 2860 | 2060 | 557 | 3024 | 1876 | 2718 |
| Notes: The dependent variable for these OLS regressions is a binary variable that is 0 if the individual does not take any decisions, and 1 if they take decisions either solely or jointly. | | | | | | |

Table A.14: Association of decisionmaking with individual and household characteristics: East Africa

|  | Kenya | | Tanzania | | Uganda | | Rwanda | |
| --- | --- | --- | --- | --- | --- | --- | --- | --- |
|  | FTF | DHS | FTF | DHS | FTF | DHS | FTF | DHS |
|  | b/se | b/se | b/se | b/se | b/se | b/se | b/se | b/se |
|  | (1) | (2) | (3) | (5) | (6) | (8) | (9) | (10) |
| Current age | 0.003^***^ | 0.001 | 0.007^***^ | 0.002 | 0.008^***^ | 0.004^***^ | 0.003^*^ | -0.005^***^ |
|  | (0.001) | (0.002) | (0.002) | (0.002) | (0.002) | (0.001) | (0.001) | (0.001) |
| Primary education or more | 0.084^*^ | 0.020 | -0.071 | 0.039 | 0.101^*^ | 0.057^***^ | 0.032 | 0.047^***^ |
|  | (0.049) | (0.033) | (0.056) | (0.033) | (0.053) | (0.017) | (0.029) | (0.013) |
| Household size | 0.002 | -0.005 | -0.021^*^ | -0.000 | 0.003 | 0.002 | 0.007 | 0.022^***^ |
|  | (0.007) | (0.005) | (0.012) | (0.007) | (0.009) | (0.004) | (0.007) | (0.004) |
| Wealth Index: 2nd quantile | -0.044 | -0.001 | -0.059 | -0.027 |  | -0.019 |  | 0.019 |
|  | (0.042) | (0.028) | (0.080) | (0.049) |  | (0.024) |  | (0.022) |
| Wealth Index: 3rd quantile | -0.026 | 0.026 | -0.103 | -0.031 |  | -0.018 |  | 0.027 |
|  | (0.050) | (0.032) | (0.088) | (0.049) |  | (0.026) |  | (0.021) |
| Wealth Index: 4th quantile | 0.072 | 0.052 | 0.018 | 0.068 |  | 0.005 |  | 0.045^*^ |
|  | (0.069) | (0.044) | (0.085) | (0.043) |  | (0.029) |  | (0.027) |
| Wealth Index: 5th quantile | 0.008 | 0.038 | -0.035 | 0.093^**^ |  | 0.029 |  | 0.024 |
|  | (0.057) | (0.049) | (0.100) | (0.042) |  | (0.032) |  | (0.027) |
| Wealth Index: 6th quantile | 0.095 | 0.104 | -0.064 | 0.013 |  | 0.080^**^ |  | 0.047 |
|  | (0.090) | (0.064) | (0.160) | (0.050) |  | (0.040) |  | (0.032) |
| Wealth Index: 7th quantile | 0.072 | 0.045 | 0.065 | 0.117^**^ |  | 0.069 |  | 0.041 |
|  | (0.087) | (0.041) | (0.108) | (0.051) |  | (0.045) |  | (0.036) |
| Language mismatch |  |  |  |  | 0.088 | -0.029 |  |  |
|  |  |  |  |  | (0.063) | (0.028) |  |  |
| Constant | 0.184^*^ | 0.131^*^ | -0.009 | 0.805^***^ | -0.196^**^ | 0.449^*^ | 0.646^***^ | -0.039 |
|  | (0.099) | (0.077) | (0.240) | (0.169) | (0.093) | (0.266) | (0.076) | (0.102) |
| Rural dummy | Yes | Yes | Yes | Yes | Yes | Yes | Yes | Yes |
| Division FE | Yes | Yes | Yes | Yes | Yes | Yes | Yes | Yes |
| Month FE | Yes | Yes | Yes | Yes | Yes | Yes | Yes | Yes |
| Interviewer FE | No | Yes | Yes | Yes | No | Yes | No | Yes |
| R-squared | 0.117 | 0.052 | 0.363 | 0.280 | 0.196 | 0.229 | 0.117 | 0.120 |
| Mean dependent variable | 0.19 | 0.27 | 0.49 | 0.57 | 0.49 | 0.66 | 0.83 | 0.69 |
| N | 1182 | 2746 | 504 | 1659 | 490 | 3998 | 658 | 5693 |
| Notes: The dependent variable for these OLS regressions is a binary variable that is 0 if the individual does not take any decisions, and 1 if they take decisions either solely or jointly. Information on asset ownership was not available in the Uganda FTF, which is why household wealth quantiles are missing. | | | | | | | | |

Table A.15: Association of decisionmaking with individual and household characteristics: Central America

|  | Haiti | | Honduras | |
| --- | --- | --- | --- | --- |
|  | FTF | DHS | FTF | DHS |
|  | b/se | b/se | b/se | b/se |
|  | (1) | (2) | (3) | (4) |
| Current age | 0.006^**^ | -0.003^**^ | 0.001 | 0.003^**^ |
|  | (0.002) | (0.001) | (0.001) | (0.001) |
| Primary education or more | -0.042 | 0.046^*^ | 0.105^***^ | 0.076^***^ |
|  | (0.062) | (0.024) | (0.030) | (0.023) |
| Household size | -0.007 | 0.007 | -0.005 | -0.004 |
|  | (0.009) | (0.005) | (0.006) | (0.005) |
| Wealth Index: 2nd quantile |  | 0.015 | -0.345^***^ | -0.045 |
|  |  | (0.037) | (0.066) | (0.032) |
| Wealth Index: 3rd quantile |  | -0.047 | -0.453^***^ | -0.022 |
|  |  | (0.034) | (0.043) | (0.035) |
| Wealth Index: 4th quantile |  | -0.009 | -0.393^***^ | -0.001 |
|  |  | (0.035) | (0.042) | (0.033) |
| Wealth Index: 5th quantile |  | -0.002 | -0.397^***^ | 0.040 |
|  |  | (0.036) | (0.044) | (0.046) |
| Wealth Index: 6th quantile |  | -0.034 | -0.325^***^ | -0.034 |
|  |  | (0.033) | (0.047) | (0.045) |
| Wealth Index: 7th quantile |  | 0.013 | -0.252^***^ | 0.042 |
|  |  | (0.038) | (0.051) | (0.048) |
| Interviewer is female |  |  | 0.028 |  |
|  |  |  | (0.030) |  |
| Constant | 0.244 | 0.931^***^ | 0.428^***^ | 0.022 |
|  | (0.284) | (0.067) | (0.096) | (0.149) |
| Rural dummy | No | No | No | No |
| Division FE | Yes | Yes | Yes | Yes |
| Month FE | Yes | Yes | Yes | Yes |
| Interviewer FE | Yes | Yes | No | Yes |
| R-squared | 0.236 | 0.192 | 0.165 | 0.083 |
| Mean dependent variable | 0.58 | 0.39 | 0.28 | 0.53 |
| N | 402 | 2583 | 1877 | 2615 |
| Notes: The dependent variable for these OLS regressions is a binary variable that is 0 if the individual does not take any decisions, and 1 if they take decisions either solely or jointly. Information on asset ownership was not available in the Haiti FTF, which is why household wealth quantiles are missing. | | | | |
